# A polymer electrolyte design enables ultralow-work-function electrode for high-performance optoelectronics

**DOI:** 10.1038/s41467-022-32651-z

**Published:** 2022-08-25

**Authors:** Bo Tong, Jinhong Du, Lichang Yin, Dingdong Zhang, Weimin Zhang, Yu Liu, Yuning Wei, Chi Liu, Yan Liang, Dong-Ming Sun, Lai-Peng Ma, Hui-Ming Cheng, Wencai Ren

**Affiliations:** 1 Shenyang National Laboratory for Materials Science, Institute of Metal Research, Chinese Academy of Sciences, Shenyang, 110016 China; 2 School of Materials Science and Engineering, University of Science and Technology of China, Shenyang, 110016 China; 3 College of Sciences, Shenyang University of Chemical Technology, Shenyang, 110142 China; 4 Shenzhen Institute of Advanced Technology, Chinese Academy of Sciences, Shenzhen, 518055 China

**Keywords:** Graphene, Lasers, LEDs and light sources, Graphene

## Abstract

Ambient solution-processed conductive materials with a sufficient low work function are essential to facilitate electron injection in electronic and optoelectronic devices but are challenging. Here, we design an electrically conducting and ambient-stable polymer electrolyte with an ultralow work function down to 2.2 eV, which arises from heavy n-doping of dissolved salts to polymer matrix. Such materials can be solution processed into uniform and smooth films on various conductors including graphene, conductive metal oxides, conducting polymers and metals to substantially improve their electron injection, enabling high-performance blue light-emitting diodes and transparent light-emitting diodes. This work provides a universal strategy to design a wide range of stable charge injection materials with tunable work function. As an example, we also synthesize a high-work-function polymer electrolyte material for high-performance solar cells.

## Abstract (web summary)

Ambient-stable solution-processed conductive materials with a low work function are essential to facilitate electron injection. Here, the authors design and synthesise polymer electrolyte with work function down to 2.2 eV for applications in high-performance light-emitting diodes and solar cells.

## Introduction

High-performance thin-film optoelectronic devices, such as perovskite solar cells (PSCs), organic solar cells (OSCs), organic light-emitting diodes (OLEDs), quantum dot (QD)-LEDs, require at least one electrode with a sufficiently low work function (*WF*) to inject electrons into the lowest unoccupied molecular orbitals (LUMO) of a given semiconductor^1–6^. Such materials are also essential for achieving ohmic contacts with the semiconductor layer in semiconductor electronic devices^2–14^. However, low-*WF* conductors such as alkaline-earth metals and reactive metal combinations are easily oxidized in the presence of ambient oxygen and water, which limits the device design and processing, especially for the solution-processed devices^5,6,15^.

An appealing alternative way to chemically reactive conductors is to modify air-stable conductors by coating a stable low-*WF* electron injection layer (EIL), which mediates charge injection and transport between conductor and the given semiconductor^2,9–11,16^. Surface dipolar polymers such as polyethylenimine ethoxylated (PEIE), branched polyethylenimine (PEI) and self-assembled dipolar molecular monolayers (SAMs) are a class of stable EIL materials^1,8–10^. They can be synthesized from solution in air and produce *WF* reduction (∆*WF*) up to 1.5 eV for a wide range of conductors by creating strong molecular and/or interface dipoles^1,10^. However, these EIL materials are limited to insulators, which would cause large barriers for electron injection and transport^16–18^. Although semiconducting conjugated polyelectrolytes are promising alternatives, they generally yield a small ∆*WF* of 0.4–0.9 eV^11,12^. Recently, a *WF* as low as 2.4 eV has been achieved by n-doping the semiconductor core of π-conjugated polyelectrolyte with multivalent anions^2,11^, whose donor strength is activated by dehydration of anion dispersion into anhydrous small ion clusters and multiplets.

Polymer electrolytes generally comprise a salt dissolved in a polymer to form polymer-salt complexes, mimicking a solid-state version of liquid electrolyte system^19,20^. In addition to the good processability and high safety, polymer electrolyte exhibits excellent electrolytic properties in terms of high ionic conductivity and low voltage polarization. Therefore, it has been widely used in batteries and supercapacitors instead of liquid electrolytes, in particular for high-performance solid-state batteries and supercapacitors^21–23^. Furthermore, polymer electrolyte can be used in dye-sensitized solar cells to improve the power conversion efficiency. Compared with liquid electrolyte, it would be able to freeze the degradation of cell performance upon time, and the regeneration of dye and the charge transfer between counter electrode can be achieved through the oxidation-reduction of redox couple (such as I^−^/I^3−^, Co^2+^/Co^3+^) in polymer electrolyte^24–26^. Importantly, the wide tunability of the salts and polymers provides a plenty of room for tailoring the properties of polymer electrolytes. Unfortunately, the known polymer electrolytes typically have excellent ionic conductivity but suffer from very poor electrical conductivity and insufficient low *WF*.

Here, we design an ambient solution-processed electrical conducting polymer electrolyte with a *WF* down to 2.2 eV, which was realized by dissolving LiClO_4_ in two-phase hybrid polysiloxane (TPHP) in acidic environment, named as TPHP(LiClO_4_). Polysiloxane matrix contains a number of oxygenated functional groups^27^ (such as C-O-Si, C = O and -OH bonds) and LiClO_4_ can be well dispersed in such matrix due to the low lattice energy^28^. Importantly, LiClO_4_ can form complex with TPHP through the coordinative bonds between Li^+^ and O atoms with lone-pair electrons. According to the orbital analysis, the LUMO and the highest occupied molecular orbital (HOMO) of TPHP monomer are mainly distributed at the C = O position and C-O-Si position, respectively. Theoretically, 1 mol TPHP can coordinate with up to 8 mol Li^+^ since 1 mol silicon-oxygen ring contains 4 mol C-O-Si bonds and 4 mol C = O bonds, which allows dissolution of a large amount of LiClO_4_ in TPHP. Such high concentration of salt is expected to have a significant impact on the percolation related properties such as electrical conductivity and Fermi energy level (*E*_F_)^29^.

## Results

### Synthesis and structure of TPHP(LiClO_4_) polymer electrolyte

**Fig. 1:**
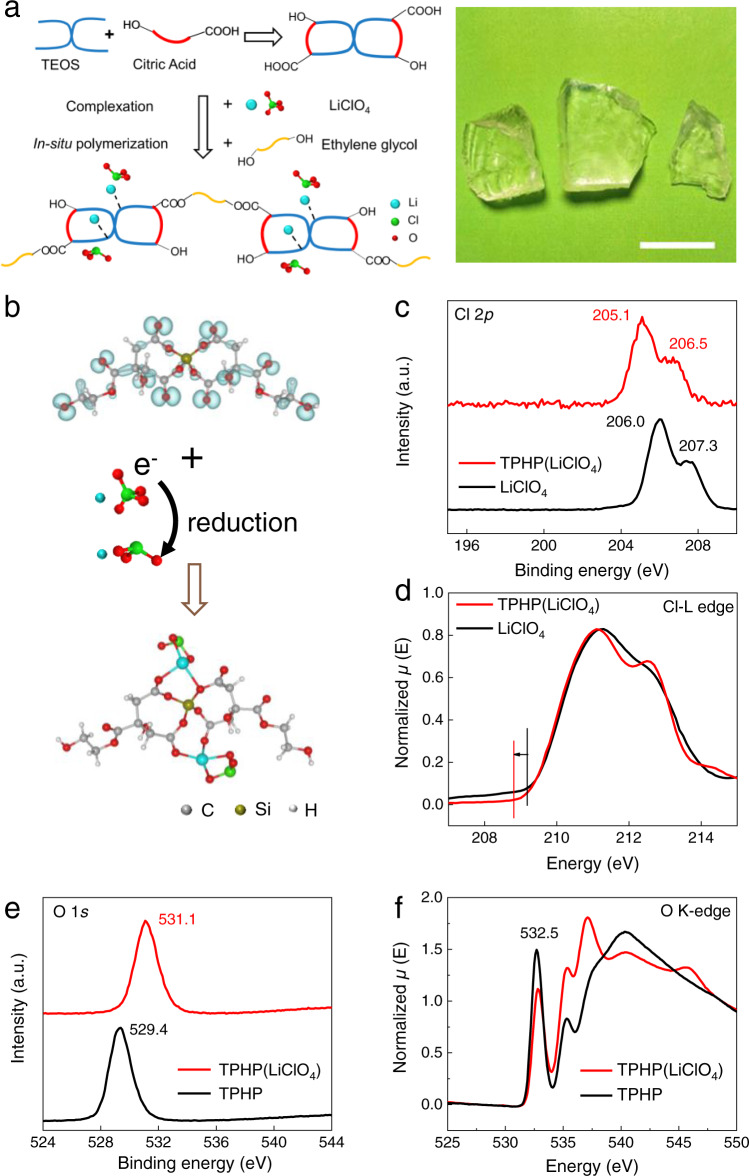
Synthesis and structure of TPHP(LiClO_4_) polymer electrolyte. **a** Schematic of the synthesis process of TPHP(LiClO_4_) (left) and photograph of the obtained bulk TPHP(LiClO_4_) polymer electrolyte (right). The scale bar is 1 cm. **b** The structure of TPHP (top) and TPHP(LiClO_4_) (bottom). The blue regions in TPHP show the HOMO orbitals, which coordinate with Li^+^ when introducing LiClO_4_. **c** Cl 2*p* XPS spectra of LiClO_4_ powder and TPHP(LiClO_4_) coatings on SiO_2_/Si substrate. **d** Cl L-edge XANES spectra of LiClO_4_ and TPHP(LiClO_4_) in the range of 207 eV to 215 eV. *µ* is the X-ray absorption coefficient at and above the absorption edge of a selected element and *E* is energy. **e** O 1 *s* XPS spectra of TPHP and TPHP(LiClO_4_) coatings on SiO_2_/Si substrate. **f** O K-edge XANES spectra of TPHP and TPHP(LiClO_4_). The mole ratio of Li^+^/TPHP in TPHP(LiClO_4_) is 2:1.

We synthesized TPHP(LiClO_4_) polymer electrolyte by a simple “complexation and in-situ polymerization” process (Fig. 1a, b and Supplementary Fig. 1). Siloxane monomer was first synthesized by the ester substitution reaction of citric acid (CA) and tetraethyl-orthosilicate (TEOS)^30^ and then complexed with LiClO_4_. TPHP(LiClO_4_) polymer electrolyte was finally formed by in-situ polymerization of siloxane monomer with ethylene glycol, followed by thermal annealing in vacuum to remove the solvents. In the above acidic reaction environment, some ClO_4_^−^ was reduced to low valence ClO_3_^−^ by redox reaction^31,32^ C_2_H_5_OH + 2ClO_4_^−^ $\mathop{\to }\limits^{{H}^{+}}$ CH_3_COOH + 2ClO_3_^−^ + H_2_O (Fig. 1a, b), which is proved by X-ray photoelectron spectroscopy (XPS) and X-ray absorption near edge structure (XANES). Note that the valence state of Cl is between +5 and +7 based on Cl 2*p* XPS (Fig. 1c). Moreover, the corresponding Cl L-edge XANES shifts towards the low-energy end of the spectra along with the increase of peak shoulder at 210−215 eV (Fig. 1d and Supplementary Fig. 2a).

Figure 1b shows the optimized structure of TPHP(LiClO_4_) polymer electrolyte obtained by density functional theory (DFT) calculation, in which Li^+^ ions form complex with TPHP through the coordinative bonds between Li^+^ and O atoms with lone-pair electrons, while ClO_3_^−^ is distributed around Li^+^. The coordination was evidenced by XPS, XANES and Fourier transform infrared (FT-IR) spectroscopy characterizations. With the introduction of LiClO_4_, the Cl 2*p* binding energies decrease, while O 1 *s* binding energy of TPHP increases in the XPS spectra (Fig. 1c, e)^33^. Meanwhile, the intensity of 532.5 eV peak corresponding to O element in the TPHP(LiClO_4_) decreases in the O K-edge XANES spectra (Fig. 1f)^34^, and the IR absorption peaks corresponding to C-O-Si and C = O stretching blue shift (Supplementary Fig. 2b).

### Electronic structure and properties of TPHP(LiClO_4_)

**Fig. 2:**
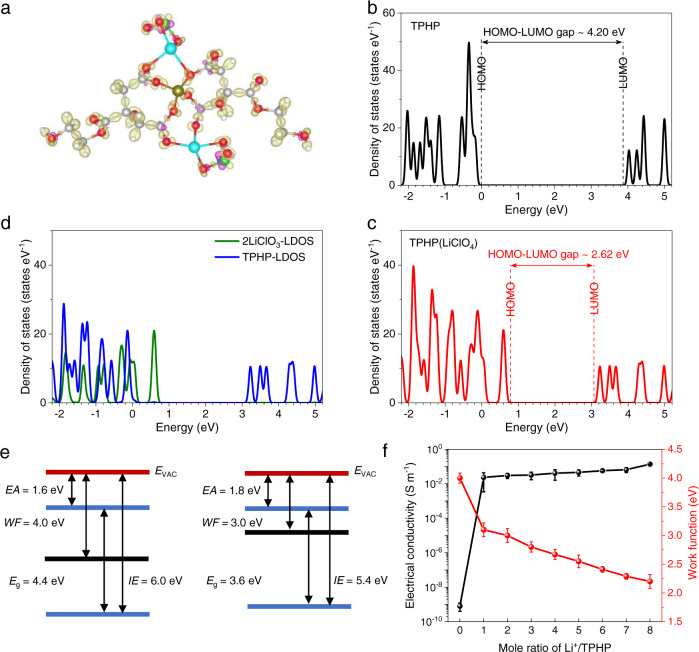
Electronic structure and properties of TPHP(LiClO_4_). **a** Charge density difference plots for TPHP(LiClO_4_). The isosurface value was set to be 0.04 e Å^−3^, the charge accumulated and depleted regions were shown in yellow and purple, respectively, clearly showing the electron transfer from ClO_3_^−^ to TPHP. **b**, **c** Calculated density of states (DOS) of TPHP (**b**) and TPHP(LiClO_4_) (**c**), in which the HOMO level was set to be aligned with *WF*. **d** Calculated local DOS (LDOS) of 2LiClO_3_ (green line) and TPHP (blue line) in TPHP(LiClO_4_). **e** Energy level alignment of TPHP (left) and TPHP(LiClO_4_) (right). *EA*, *E*_g_, *IE* and *E*_VAC_ represent electron affinity, bandgap, ionization energy and vacuum energy level, respectively. The Li^+^/TPHP mole ratio is 2:1 for TPHP(LiClO_4_) in **a**–**e**. **f** Dependence of electrical conductivity and *WF* of TPHP(LiClO_4_) on the concentration of LiClO_4_ in TPHP. The electrical conductivities of TPHP(LiClO_4_) were obtained based on impedance measurements (Supplementary Fig. 5b, c) and those of TPHP were measured using a high resistance meter.

The stability of ClO_3_^−^ with triangular cone structure is not as good as ClO_4_^−^ with tetrahedral structure, and thus the intermediate valance Cl ions in ClO_3_^−^ tend to lose electrons. Meanwhile, the formation of Li^+^-TPHP coordination bonds provides charge transfer channels from ClO_3_^−^ to TPHP. These two effects lead to n-doping of TPHP and consequently modify its *WF*, bandgap and conductivity. Bader charge analysis shows that 0.07 mol electrons are transferred from ClO_3_^−^ to TPHP for TPHP(LiClO_4_) with Li^+^/TPHP mole ratio of 2:1 (Fig. 2a), confirming the n-doping effect of ClO_3_^−^ on TPHP. The electronic structure calculations show that the *E*_F_ and bandgap of TPHP are −6.23 eV and 4.2 eV, respectively, while they changed to −5.42 eV and 2.62 eV for TPHP(LiClO_4_), corresponding to a *WF* reduction of 0.81 eV and bandgap reduction of 1.58 eV (Fig. 2b, c). Further analyses indicate that the HOMO change is dominantly from the contribution of LiClO_3_ (Fig. 2d), providing another evidence that ClO_3_^−^ acts as electron donor for the n-doped TPHP.

Inverse photoemission spectroscopy (IPES) and ultraviolet photoemission spectroscopy (UPS) measurements were performed to characterize the *WF* and bandgap, during which a very low radiation energy and fast scanning were used to make sure that TPHP(LiClO_4_) was not damaged (Supplementary Fig. 3)^35^. The results show that *WF* and bandgap of TPHP is reduced from 4.0 to 3.0 eV and from 4.4 to 3.6 eV, respectively, when introducing LiClO_4_ with a mole ratio of Li^+^/TPHP of 2:1 (Fig. 2e, and Supplementary Fig. 4). These experimental results are consistent with the above calculation results. Meanwhile, the electrical conductivity is substantially increased from ~10^−10^ to ~10^−2 ^S·m^−1^ (Fig. 2f and Supplementary Table 1), which is significantly higher than those of dipolar polymer EIL, typically around 10^−7^ S·m^−1^, as well as known polymer electrolytes (10^−5^ − 10^−6 ^S·m^−1^)^19–22^. Moreover, the *WF* is gradually reduced while conductivity is further improved with the increase of mole ratio of Li^+^/TPHP (Fig. 2f, Supplementary Fig. 5 and Supplementary Table 2), implying the enhancement of doping effect of LiClO_3_ with the increase of ClO_3_^−^ content and coordination number. Notably an ultralow *WF* of 2.2 eV along with a high electrical conductivity over 10^−2^ S·m^−1^ were achieved at a mole ratio of Li^+^/TPHP of 8:1.

### *WF* modification of various conductors with TPHP(LiClO_4_)

Another important feature of TPHP(LiClO_4_) is that it is well suited for solution processing. It has good solubility in common organic solvents, such as ethanol, dimethyl sulfoxide (DMSO), N-methylprolinodone (NMP), isopropanol (IPA) and N, N-dimethylformamide (DMF), even with a high mole ratio of Li^+^/TPHP of 8:1 (Supplementary Fig. 6). Importantly, the coordination between Li^+^ and TPHP stabilizes LiClO_4_/LiClO_3_ and suppresses its dendrite crystallization/accumulation. Thus, the TPHP(LiClO_4_) solution is air stable, showing no change even after 8 months (Supplementary Fig. 7). In addition, TPHP(LiClO_4_) is an amphiphilic material, which includes hydrophobic polymer chain and hydrophilic carboxyl groups. Its solution shows good wettability towards a wide range of substrates such as graphene/polyethylene terephthalate (PET), indium tin oxides (ITO) glass, Cu, SiO_2_/Si and quartz (Supplementary Fig. 8).

**Fig. 3:**
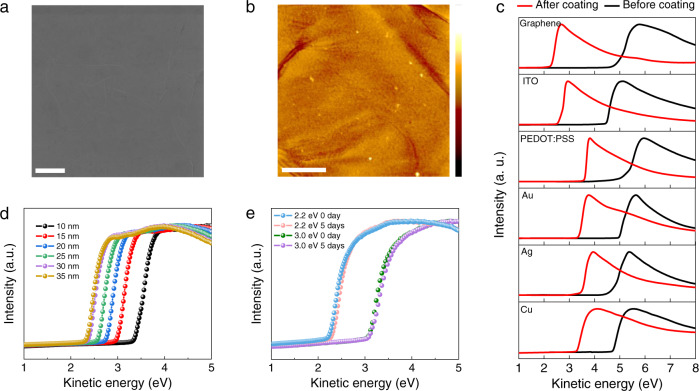
*WF* modification of various conductors with TPHP(LiClO_4_). **a**, **b** Helium ion microscope (HIM) (**a**) and atomic force microscope (AFM) (**b**) images of TPHP(LiClO_4_)-coated graphene on thermal release tape (TRT) substrate. The scale bars in (**a**) and (**b**) are 1 μm, and height scale in (**b**) is from −5 to 5 nm. **c** Photoemission cutoff obtained via UPS for graphene, ITO, PEDOT: PSS PH1000, Au, Ag and Cu conductors before (black plots) and after (red plots) coating 30-nm-thick 2.2 eV TPHP(LiClO_4_). **d** UPS spectra of different thickness of 2.2 eV TPHP(LiClO_4_)-coated graphene, showing continuous *WF* reduction with coating thickness until 30 nm. **e** UPS spectra of 30-nm-thick TPHP(LiClO_4_)-coated graphene with *WF* of 2.2 eV and 3.0 eV, respectively, before and after standing in air for 5 days.

The above features enable uniform and smooth TPHP(LiClO_4_) coatings on various conductors and semiconductors. For instance, the TPHP(LiClO_4_)-coated transferred chemical vapor deposition (CVD)-grown graphene shows a root mean square (*RMS*) roughness of ~0.75 nm, better than that without coating (Fig. 3a, b and Supplementary Fig. 9), and uniform sheet resistance (*R*_s_) distribution (Supplementary Fig. 10). TPHP(LiClO_4_)-coated fused silica has an *RMS* roughness of ~0.95 nm from ethanol solution and 0.43 nm from NMP solution (Supplementary Fig. 11), both of which are much smaller than those of reported n-doping π-conjugated polyelectrolyte-coated fused silica (~3.1 nm). Furthermore, TPHP(LiClO_4_) coating can well bind the substrates (Supplementary Fig. 12), which is essential for achieving low-*WF* conductors and improving contact.

We coated TPHP(LiClO_4_) on various conductors, including graphene, ITO, poly(3,4-ethylenedioxythiophene)-poly(styrenesulfonate) (PEDOT:PSS) and metals (Au, Ag, Cu) to explore its potential as EIL (Fig. 3c and Supplementary Fig. 13). Supplementary Table 3 summarizes the *WF*s of conductors before and after coating 2.2 eV TPHP(LiClO_4_) and the corresponding ∆*WF*s. The results suggest the universality of TPHP(LiClO_4_) to reduce the *WF* of conductors, among which TPHP(LiClO_4_)-coated graphene shows the most noticeable *WF* reduction. Moreover, *WF* reduction of TPHP(LiClO_4_)-coated conductors depends on the *WF* of TPHP(LiClO_4_) and increases with the thickness of the coating (Fig. 3c, d and Supplementary Fig. 13). The *WF* of 2.2 eV TPHP(LiClO_4_)-coated graphene reaches that of TPHP(LiClO_4_), as the thickness of TPHP(LiClO_4_) coating is increased to 30 nm (Fig. 3d). Such ∆*WF* of 2.45 eV is much larger than those induced by the reported EIL materials^1,2^.

Importantly, TPHP(LiClO_4_) is fairly stable under ambient conditions compared to the reported low-*WF* EIL materials. We measured the ambient stability of 30-nm-thick TPHP(LiClO_4_)-coated graphene with *WF* of 3.0 eV and 2.2 eV, respectively. As shown in Fig. 3e, the former shows only ~0.01 eV increase in *WF* after standing in air for 5 days. This is significantly better than LiF with similar *WF* (~3.0−3.4 eV), which fails within 5 mins even under nitrogen atmosphere^6^. Even for the TPHP(LiClO_4_)-coated graphene with an ultralow *WF* of 2.2 eV, it only shows ~0.05 eV increase in *WF* after standing in air for 5 days. On the one hand, the good ambient stability of TPHP(LiClO_4_) is attributed to the coordination between Li^+^ and O in TPHP matrix, which stabilizes the chemically active Li^+^. On the other hand, the TPHP polymer substrate can act as a skin to protect the encapsulated Li^+^ from catalytic reaction with water and oxygen in air.

The *WF* reduction of conductors induced by semiconducting TPHP(LiClO_4_) coating is attributed to their Schottky contact caused by different *WF*, which leads to charge transfer and consequently *E*_F_ change^36^. Driven by *E*_F_ alignment, energy band bending occurs at TPHP(LiClO_4_)/conductor interface, creating a space charge region (Supplementary Fig. 14a). As a result, the *WF* of conductor continuously decreases with TPHP(LiClO_4_) thickness until the space charge region width (Supplementary Fig. 14a). In addition, the *E*_F_ change strongly depends on the density of states (DOS) of conductors, and thus *WF* reductions varies with conductors. As shown in Supplementary Fig. 14b and Supplementary Table 3, the electron transfer induces a very small *E*_F_ upshift for conductors with high DOS, such as Au, Ag and Cu, while *E*_F_ is significantly increased for conductors with low DOS such as graphene, ITO and PEDOT:PSS. The large Dirac voltage (*V*_Dirac_) shift from +22.3 V to −16.9 V (Supplementary Fig. 14c, d) gives another evidence of substantial electron transfer and upshift of *E*_F_ of graphene^18^, along with energy band bending, which enable graphene the most noticeable *WF* reduction.

Besides reducing *WF*, the substantial electron transfer from TPHP(LiClO_4_) coating also greatly improves the electrical conductance of conductors. For instance, compared to graphene, TPHP(LiClO_4_)-coated graphene shows about 50% decrease in *R*_s_ (Supplementary Fig. 15a and Supplementary Table 4). The conductance is fairly stable and only a slight *R*_s_ increase by 10% was observed after two months in air (Supplementary Fig. 16). This is in contrast to the insulating dipole polymers like PEIE, only 10-nm-thick coating of which leads to about 20% increase in *R*_s_. In addition, TPHP(LiClO_4_) might also act as an anti-reflection film to balance the optical transmittance (*Tr*) loss induced by coating^37^. As a result, the TPHP(LiClO_4_)-coated graphene becomes more transparent with the increase of coating thickness (Supplementary Fig. 15b, c and Supplementary Table 4).

To prove the role of low-*WF* TPHP(LiClO_4_) in improving electron injection of conductors^38,39^, we fabricated Schottky diodes with Al and Au as asymmetrical electrodes and diketopyrrolopyrrole-thieno[3,2-b]thiophene (DPPT-TT) as n-type semiconductor (Supplementary Fig. 17a). The HOMO and LUMO energy levels of DPPT-TT are 5.15 eV and 3.5 eV, respectively, which are similar to the *WF* of Au (5.1 eV) and Al (3.8 eV). This allows a good contact of DPPT-TT with Al when a positive bias is applied (Supplementary Fig. 17b). However, when applying a negative bias, the electron-injection efficiency is greatly reduced, leading to a very small leakage current (Supplementary Fig. 17b, c). Notably, the leakage current at negative bias is increased by ~100 times with coating 20-nm-thick 2.2 eV TPHP(LiClO_4_) EIL, suggesting that the injection efficiency of electrons from Au to DPPT-TT is effectively improved. Because the *WF* of TPHP(LiClO_4_)-coated conductor changes with the *WF* of TPHP(LiClO_4_), the leakage current can be tuned accordingly (Supplementary Fig. 17d).

### High-performance blue QD-LEDs enabled by TPHP(LiClO_4_) EIL

**Fig. 4:**
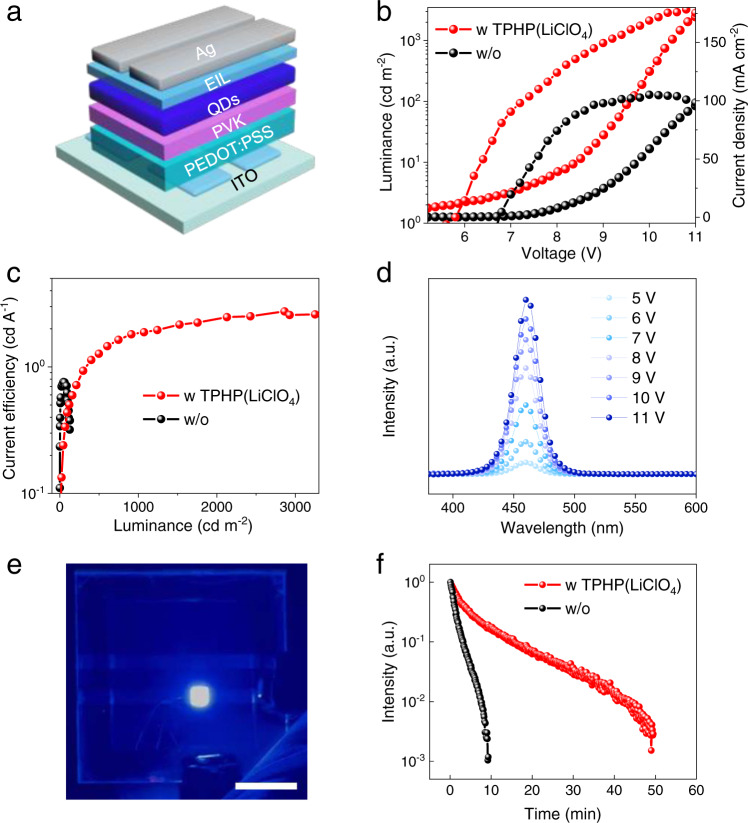
High-performance blue QD-LEDs enabled by TPHP(LiClO_4_) EIL. **a** Device structure. **b**, **c** *J-V*-*L* (**b**) and *CE*-*L* (**c**) characteristics for the devices with and without 20-nm-thick 2.2 eV TPHP(LiClO_4_) EIL. **d** EL spectra of blue QD-LED with 20-nm-thick 2.2 eV TPHP(LiClO_4_) EIL. **e** A photograph of a lighted blue QD-LED. Scale bar is 1 cm. **f** Comparison of normalized luminance of the QD-LEDs with and without TPHP(LiClO_4_) as a function of operating time at a constant current density and an initial luminance of ~100 cd m^−2^ under ambient conditions.

Blue LEDs are the core elements indispensable for solid-state lighting and full-color display technologies^40^. Low-*WF* EIL is highly desired for blue LEDs due to the relatively wide bandgap of blue emitting layer, which can substantially improve the electron injection from cathode to blue emitting layer. We fabricated solution-processed blue QD-LEDs using 20-nm-thick 2.2 eV TPHP(LiClO_4_)-coated Ag as top cathode (Fig. 4a). As a result of reduced *WF*, the interface resistance between Ag and blue QDs is reduced (Supplementary Fig. 18), which enables much better device performances. Compared to the devices without TPHP(LiClO_4_) EIL, *V*_turn-on_ decreases from 6.6 to 5.6 V, maximum current efficiency (*CE*_MAX_) increases from 0.80 to 2.77 cd A^−1^, and maximum luminance (*L*_MAX_) substantially increases from 135.3 to 2525 cd m^−2^ (Fig. 4b, c). Notably, the *L*_MAX_ is increased by ∼20 times at the same voltage. More importantly, the *J-V* characteristics is independent of the thickness of TPHP(LiClO_4_) EIL up to 50 nm (Supplementary Fig. 19), which confirms that the EIL has a sufficiently high electrical conductivity^2^.

Furthermore, the blue QD-LEDs with TPHP(LiClO_4_) EIL have much better stability than those without EIL under ambient conditions, showing identical electroluminescence (EL) peaks at different voltage and largely improved lifetime (Fig. 4d–f). The device degradation originates from electron accumulation at Ag/blue QDs interface during operation, which results in heat accumulation and consequently oxidizes the blue-QDs^41,42^. The low *WF* of TPHP(LiClO_4_) improves the electron injection from Ag to blue-QDs and consequently reduces electron accumulation, leading to largely improved stability. These results demonstrate the significant role of low-*WF* TPHP(LiClO_4_) in improving the performance of blue LEDs. Considering the good stability of TPHP(LiClO_4_) itself (Fig. 3e), we expect that both the lifetime and operation stability of devices can be greatly improved if they are encapsulated to minimize the detrimental effects of oxygen and moisture in air on QDs.

### Transparent QD-LED and OLED with TPHP(LiClO_4_) EIL

Transparent lighting and display devices offer new opportunities for wearable electronics, virtual reality and augment reality, etc., in which both bottom and top electrodes are transparent^43^. However, the top electrode is much more difficult to be realized compared with the bottom one. Except for good conductance, high transparency and low *WF*, as the final processed layer, the processing of the top electrode should not affect the pre-fabricated underlying emission layers. The magnetron sputtering deposition for ITO and indium zinc oxide (IZO) and the high surface roughness for silver nanowires (Ag NWs) can destroy the underlying layers, leading to poor device performances^43,44^. Highly conductive CVD graphene is an appealing top electrode, but it needs to be transferred to the underlying electron transport layer (ETL) followed by peeling off from the transfer support to ensure high transparency, during which the graphene can be easily broken^45,46^.

**Fig. 5:**
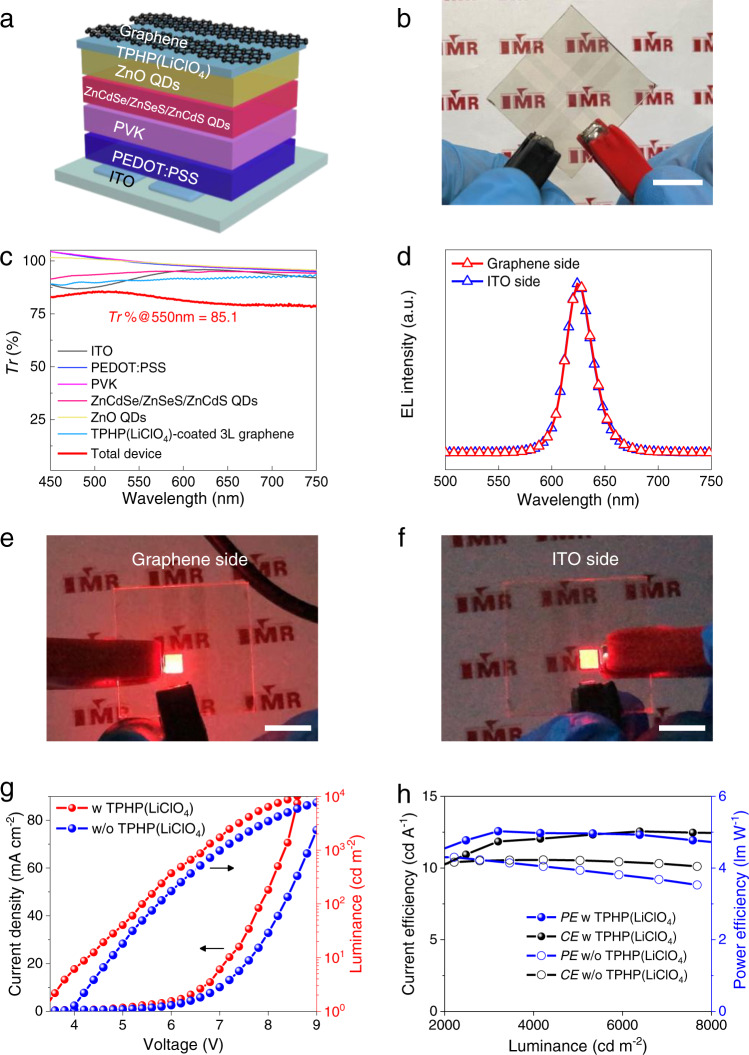
Transparent red QD-LED with graphene top electrode and TPHP(LiClO_4_) EIL. **a**, **b** Device structure (**a**) and photograph (**b**) of QD-LED. **c** *Tr* spectra of each layer and the total QD-LED device. **d** EL spectra from graphene and ITO sides. **e**, **f** Photograph of a lighted QD-LED on graphene (**e**) and ITO (**f**) sides. The scale bars in (**b**, **e** and **f**) are 1 cm. **g**, **h** Typical *J-V*-*L* (**g**), *CE*-*L* and *PE*-*L* (**h**) characteristics of devices with and without TPHP(LiClO_4_) EIL. The TPHP(LiClO_4_) EILs used in all the devices have a *WF* of 3.0 eV and thickness of 25 nm.

The excellent combined properties of TPHP(LiClO_4_) EIL enable the fabrication of various high-performance transparent optoelectronics using graphene as top electrode. As the first example, we fabricated a transparent red QD-LED (Fig. 5a, b), by coating QD-LED functional layers onto ITO glass and then transferring a TPHP(LiClO_4_)-coated graphene on the top as cathode (Supplementary Fig. 20). The good binding nature of TPHP(LiClO_4_) EIL facilitates graphene transfer and contact with ETL, and 3 layers are enough to ensure sufficient low *R*_s_. The resulting red QD-LED is highly transparent with *Tr* of ~85.1% (Fig. 5b, c), which is much higher than those of the reported transparent red QD-LED devices (less than 80%, Supplementary Table 5) and satisfies the requirement for transparent displays. Importantly, among 32 fabricated devices, ~90% devices can be lit up and exhibit uniform luminance across the whole lighting area with identical EL spectra from both sides (Fig. 5d–f, Supplementary Fig. 21a,  22 and Supplementary Movie 1). In contrast, without using EIL, only ~30% devices could be partially lit up due to the breaking of graphene (Supplementary Fig. 21b,  23).

Furthermore, the low *WF* of TPHP(LiClO_4_)-coated graphene enables ohmic contact with ZnO ETL, which improves the electron injection and recombination with holes (Supplementary Fig. 24 and Supplementary Table 6)^47^. Thus, our red QD-LEDs show high performance with a low *V*_turn-on_ down to 3.4 V and a high *L*_MAX_ up to ~13,000 cd m^−2^ at 9 V (Fig. 5g and Supplementary Fig. 25). The average *CE*_MAX_ and maximum power efficiency (*PE*_MAX_) can reach 12.55 cd A^−1^ and 5.03 lm W^−1^, respectively, both of which are much higher than those of devices without EIL (Fig. 5h and Supplementary Fig. 25) and nearly 10 times higher than those of the reported red transparent QD-LED devices with Ag NWs, or CVD graphene hybrids as top electrodes (Supplementary Table 5). In addition, our devices show much better stability than those of devices without EIL and with stable PEIE under ambient conditions (Supplementary Fig. 26), and they can run multiple times below 9 V without transient effects during operation (Supplementary Fig. 27, 28)^48^.

Air-stable, low-*WF* top electrodes are also highly desired for other types of transparent optoelectronic devices. As another example, we fabricated a transparent green OLED by using ITO bottom electrode, graphene top electrode and TPHP(LiClO_4_) EIL, which also shows uniform luminescence on both sides (Supplementary Fig. 29, Supplementary Movie 2). The OLED exhibits a high *Tr* of ~85%, *L*_MAX_ of ~9800 cd m^−2^ at 11 V, *CE*_MAX_ of 44.5 cd A^−1^ and *PE*_MAX_ of 19.9 lm W^−1^, all of which are the best among the reported transparent green OLEDs (Supplementary Table 7). These results highlight the universal role of TPHP(LiClO_4_) in fabricating low-*WF* top electrodes for various transparent optoelectronic devices.

## Discussion

Importantly, the polymer electrolyte can be designed for high-*WF* materials by choosing appropriate salts. For instance, we replaced LiClO_4_ with CuCl_2_ to synthesize a high-*WF* TPHP(CuCl_2_) polymer electrolyte with a *WF* of 5.35 eV and electrical conductivity of 2.28 × 10^−2 ^S·m^−1^. After coating onto graphene, the *WF* of graphene increases from 4.65 to 5.20 eV (Supplementary Fig. 30a), along with a low *R*_s_ of 330 Ω·sq^−1^ and a high *Tr* of 97.4% (Supplementary Fig. 30b, c). The OSC with TPHP(CuCl_2_) as hole injection layer (HIL) and graphene as bottom anode achieves a high efficiency of 5.5% (Supplementary Fig. 30d–f and Supplementary Table 8). We further demonstrate the use of TPHP(LiClO_4_) EIL and TPHP(CuCl_2_) HIL together for improving the performances of optoelectronic devices. It can be found that the efficiencies of transparent QD-LED are further improved with the addition of TPHP(CuCl_2_) HIL and PSCs with TPHP(CuCl_2_) HIL and TPHP(LiClO_4_) EIL achieve a high efficiency of 19.77% (Supplementary Fig. 31 and Supplementary Table 9). Considering the availability of diverse salt ions and polymers, this polymer electrolyte strategy opens up the possibilities to design a wide range of charge injection materials with tunable properties that could not be achieved with the existing materials, which will greatly booster the applications of electronic and optoelectronic devices.

## Methods

### Materials

CA (anhydrous, 99%), TEOS (anhydrous, 99%), LiClO_4_ (anhydrous, >98%), chlorobenzene, SnO_2_ colloid precursor (15% in H_2_O colloidal dispersion) and CsI (99.99%) were purchased from Alfa Aesar China Chemical Co., Ltd. Ethylene glycol, ethanol and octane were purchased from Sinopharm Chemical Reagent Co., Ltd. Blue ZnCdS/ZnS, red ZnCdSe/ZnSeS/ZnCdS and ZnO QDs for QD-LED were purchased from Poly Opto-Electronics Ltd. DPPT-TT was purchased from Xi’an Qiyue Biotechnology Co., Ltd.

PEDOT:PSS (AI 4083), PEDOT:PSS PH1000, poly(9-vinlycarbazole) (PVK), formamidinium iodide (FAI), methylammonium bromide (MABr), octylamine hydrobromide (OABr), PbI_2_ and PbBr_2_ were purchased from Xi’an Polymer Light Technology Corp. Spiro-OMeTAD (99.5%), 4-tert-butylpyridine (99.90%), lithium bis(trifluoromethylsulphonyl) imide (99.95%), and FK209 Co(III) TFSI salt (98%) were purchased from Dyesol. MoO_3_, di-[4-(N,N-ditolyl-amino)-phenyl]cyclohexane (TAPC), bis(2-phenylpyridine)(acetylacetonate) iridium(III) [Ir(ppy)_2_(acac)], bis[2-(2-hydroxyphenyl)-pyridine]beryllium (Bepp_2_), bathophenanthroline (Bphen) and LiF for OLED were purchased from Jilin OLED Material Tech Co., Ltd. ITO glass was purchased from Advanced Election Technology CO., Ltd. Copper foil, gold foil and aluminum particles were purchased from Aluminum Corporation of China. Monolayer graphene films on PET and SiO_2_/Si substrates were obtained by CVD growth on copper foil followed by transferring with poly(methyl methacrylate)/rosin (PMMA/rosin) double support layer^49^. The graphene films on TRT substrate were purchased from Sixth Element (Changzhou) Materials Technology Co., Ltd.

### Synthesis of TPHP(LiClO_4_) and its coating on various conductors

First, 0.2 mol CA was dissolved in 100 ml ethanol under stirring at room temperature. Then, 0.1 mol TEOS was mixed to form siloxane monomer by ester substitution reaction with CA. After that, LiClO_4_ was dissolved sufficiently, during which Li^+^ formed complexation with O atoms of siloxane. Finally, 0.32 mol ethylene glycol was added, and the resulting solution was heated to ~60 °C for 6 h to promote the in-situ polymerization reaction to form TPHP(LiClO_4_) polymer electrolyte gel, which was further annealed in vacuum at 100 °C overnight to obtain dried TPHP(LiClO_4_) samples.

To fabricate TPHP(LiClO_4_) film on conductors, we re-dissolved the dried TPHP(LiClO_4_) in ethanol to 3–5 mg ml^−1^. Then, spin coating was used to deposit TPHP(LiClO_4_) on different conductors, such as graphene, ITO and PEDOT:PSS, followed by drying at 60 °C for 2 h, which is enough for obtaining dried TPHP(LiClO_4_) film (Supplementary Fig. 32). The thickness of the TPHP(LiClO_4_) EIL film was adjusted by the spin coating rate (3000–5000 rpm).

### Structure and property characterizations of TPHP(LiClO_4_) EIL and TPHP(LiClO_4_)-coated conductors

Optical microscope (OM, Nikon Eclipse LV100) and HIM (Zeiss Orion NanoFab) were used to characterize the morphology of graphene and TPHP(LiClO_4_)-coated graphene on SiO_2_/Si and TRT substrate. The thickness of TPHP(LiClO_4_) coating was measured by KLA-Tencor P7 Surface Profiler on glass substrate. AFM (Bruker Multimode 8, tapping mode) was used to characterize the *RMS* roughness of graphene and TPHP(LiClO_4_)-coated graphene on TRT, and TPHP(LiClO_4_)-coated fused silica.

XPS (ESCALAB 250 instrument with Al Kα and He I radiation sources) was used to characterize the coordination between the Li^+^ and O of TPHP on a SiO_2_/Si substrate. The XPS spectra were fitted using the XPS peak 4.1 software in which a Shirley background was assumed. The oxide K-edge (525.5 eV) and Cl L-edge XANES spectroscopy measurements were performed at the beamline (4B7A) of Argonne National Laboratory. FT-IR absorption spectra of TPHP and TPHP (LiClO_4_) were tested by FT-IR (Shimazu IRAffinity − 1 S Japan) ranging from 400 cm^−1^ to 2000 cm^−1^ using air as the background. To this end, a small amount of TPHP or TPHP(LiClO_4_) solid was mixed with KBr and grinded into thin sheets. The free-standing TPHP and TPHP(LiClO_4_) membranes with a radius of 5 mm and the thickness of 5 mm were cured from polymer solution. The conductivities of TPHP membranes were measured by high resistance meter (Keithley 6517B). The impedance measurements of TPHP(LiClO_4_) membranes were carried out by using an Autolab electrochemical workstation (PGSTAT204) with tunable frequency ranging from 1 Hz to 1 MHz and an alternating potential of 100 mV. The conductivities of TPHP(LiClO_4_) films dried at different temperatures on SiO_2_/Si were also characterized by Keithley source measurement units (Keithley 2450). We first spin coated TPHP(LiClO_4_) films on SiO_2_/Si and dried at 60 °C, 120 °C or 150 °C. After that, two parallel Ag electrodes were deposited on the surface of TPHP(LiClO_4_) film by vacuum evaporation. We then measured the *I*-*V* curves and calculated the electrical conductivities (*σ*) of TPHP(LiClO_4_) films using the equation *σ* = *I* × *L*/(*V* × *W* × *T*), where *T* is the thickness of TPHP(LiClO_4_), *W* is the length of the electrode, *L* is the distance between the two electrodes^50^.

UPS measurements were performed on a PHI VersaProbe III instrument with He I 21.2 eV at 80.0 W and the beam diameter was 5.0 μm. All the measurements of the onset of photoemission were performed using standard procedures to determine the *WF*. A −5 V bias was applied onto the sample to separate the secondary electron cut-off sides, and the *E*_F_ level was calibrated using a standard Pt sample. IPES measurements of TPHP and TPHP(LiClO_4_) on Au substrate were carried out in PHI VersaProbe III mode with a resolution of 0.04 eV. Each sample was measured 12 times, *E*_VAC_ and *EA* were obtained from the average values. *E*_F_ and *IE* references were established by UPS and IPES measurements using an air-exposed Au foil surface.

*R*_s_ of graphene on PET substrate with different thickness of TPHP(LiClO_4_) coating was measured by a 4-probe resistivity measurement system (Guangzhou 4-probe tech., RTS-9, China). To evaluate the uniformity of TPHP(LiClO_4_)-coated graphene films on PET, we divided 5 × 5 cm^2^ samples into 25 equal areas to measure their *R*_s_. To obtain *Tr* of graphene films coated with different thickness of TPHP(LiClO_4_), they were transferred onto quartz substrate and measured with a UV-vis-NIR spectrometer (Varian Cary 5000), which avoid the interference fringes that were usually observed for the graphene on PET substrate.

Contact angles of TPHP(LiClO_4_) solution on different substrates were characterized by Biolin optical contact angle measuring instrument. Graphene-based transistors were fabricated as follows to evaluate the electron transfer and *E*_F_ change induced by TPHP(LiClO_4_). Graphene film was first transferred onto the SiO_2_ (280 nm)/Si substrate with pre-patterned Ti/Au as the source/drain electrodes and pre-deposited Au film as the bottom gate, and then patterned by using standard photolithography and oxygen plasma etching to fabricate transistor. The electrical properties of the devices were measured by using a semiconductor analyzer (Agilent, B1500A) under ambient conditions.

### Density functional theory calculations

DFT calculations were performed via the Vienna Ab initio Simulation package (VASP)^51^. The projector augmented wave (PAW) method^52^ and the Perdew-Burke-Ernzerhof (PBE) functional within the generalized gradient approximation^53^ were used to describe the electron-ion interactions and the exchange-correlation interactions, respectively. The kinetic energy cutoff of plane wave was set to be 520 eV and the convergence criterion for the residual forces and total energies were set to be 0.01 eV Å^−1^ and 10^−6^ eV, respectively. For simplicity, a monomer of TPHP consisting of 20 H, 16 C, 16 O and 1 Si atoms was considered to study the doping effect of LiClO_3_ on the TPHP. The empirical correction in Grimme’s method (DFT+D3) was adopted to describe the van der Waals interaction^54^. A 30 × 21 × 20 Å^3^ supercell with one TPHP monomer (C_16_H_20_SiO_16_) was constructed in order to minimize the interactions between periodic images. For this large supercell, only the Gamma-point was used to sample the Brillouin zone for all calculations. Bader charge calculation was performed to analyze the charge population and charge transfer^55^.

### Fabrication and characterizations of various devices

#### Schottky diodes

We first deposited 100-nm-thick Al array electrodes on glass substrate through a shadow mask to provide a channel (width, 100 µm; length, 5 mm). After that, DPPT-TT (8 mg mL^−1^ in chlorobenzene) was spin-coated onto their surface at 4000 rpm for 45 s and heated at 120 °C for 15 min in a nitrogen-filled glovebox. Finally, 50-nm-thick Au array electrodes were vacuum deposited through a shadow mask to obtain the control device without EIL. To fabricate devices with EIL, we spin-coated TPHP(LiClO_4_) (8–10 mg ml^−1^) at 3000–5000 rpm before depositing Au electrode.

*V*-*I* characteristics of the devices were characterized by Keithley source measurement units (Keithley 2450) under ambient conditions without encapsulation. During measurements, Al was connected as negative electrode and Au as positive electrode. The whole measurement process was under dark conditions. The applied voltage was from −5 V to +5 V and the step voltage was 0.2 V. Each device was measured 5 times to ensure the repeatability of the curves.

#### Blue QD-LED

First, ITO anodes were cleaned by deionized water, acetone and alcohol in sequence, followed by oxygen plasma treatment for 15 min. Then, PEDOT:PSS was spin-coated on ITO at 3500 rpm for 60 s and heated at 150 °C for 10 min in air. After that, we successively spin-coated PVK (10 mg mL^−1^ in chlorobenzene), ZnCdS/ZnS QDs (20 mg mL^−1^ in normal octane) at 4000 rpm for 45 s, 2500 rpm for 60 s and then heated at 120 °C for 15 min, 100 °C for 10 min, respectively, in a nitrogen-filled glovebox. Finally, 130-nm-thick Ag array electrodes were vacuum deposited through a shadow mask to obtain the control device without EIL. To fabricate the devices with EIL, we spin-coated TPHP(LiClO_4_) (8–10 mg ml^−1^) at 3000–5000 rpm before depositing Ag electrode.

*J-V*-*L* characteristics of the devices were characterized by Keithley source measurement units (Keithley 2450) and spectrophotometer (PR-655, Photo Research, Inc.) with a calibrated silicon photodiode under ambient conditions without encapsulation. Impedance spectroscopy was conducted with a computer-controlled programmable Autolab Workstation (PGSTAT204) and FRA hardware (FRA32M). The sweeping bias voltage was +3 V, and sweeping frequency from 1000 Hz to 1,000,000 Hz. All measurements were carried out with a signal of amplitude 500 mV. The stability of devices was measured with initial luminance of 100 cd m^−2^ and a constant current under ambient conditions without encapsulation, and the luminance was collected every 500 ms during measurements.

#### Transparent QD-LEDs

Supplementary Fig. 20 shows the fabrication process of the transparent QD-LED by solution method. First, ITO anodes were cleaned by deionized water, acetone and alcohol in sequence, followed by oxygen plasma treatment for 15 mins. Then, PEDOT:PSS was spin-coated on ITO at 3500 rpm for 60 s and heated at 150 °C for 10 min in air. After that, we successively spin-coated the PVK (8–10 mg mL^−1^ in chlorobenzene), ZnCdSe/ZnSeS/ZnCdS QDs (12 mg mL^−1^ in normal octane), ZnO QDs (30 mg mL^−1^ in ethanol) at 4000 rpm for 45 s, 3000 rpm for 45 s, 2500 rpm for 45 s and heated at 110 °C for 20 min, 100 °C for 10 min, 100 °C for 15 min, respectively, in a nitrogen-filled glovebox. Finally, TPHP(LiClO_4_)-coated 3 L graphene on TRT substrate was laminated on the top of ZnO QDs ETL. After keeping at 60 °C for 5–10 mins, TRT sheet was peeled off and the transparent QD-LED with pure graphene as top cathode was obtained. For comparison, graphene without TPHP(LiClO_4_) coating was also used to fabricate QD-LED with the same device structure. 32 devices were fabricated for each to evaluate the device yield and reproducibility.

*Tr*s of the devices were measured by a UV-vis-NIR spectrometer (Agilent Model Cary 5E). *J-V*-*L* characteristics and impedance spectroscopy measurements were conducted same as those for blue LEDs. The stability of devices was measured with an initial luminance of 1000 cd m^−2^. The computer-controlled programmable Autolab Workstation was used to measure the transient effect during charging and discharging under a constant transient current (0.1 mA) with sweeping rate from 0.1 to 0.6 s per cycle. Multiple cycles tests were conducted for each sweeping rate. The turn-on time and turn-off time of devices were determined by charging from 10% to 90% and discharging from 90% to 10% in the voltage waveform, respectively.

#### Transparent OLEDs

For transparent OLED fabrication, the cleaned ITO anodes on glass were loaded into a high vacuum chamber to successively deposit 10 nm MoO_x_, 60 nm TAPC, 10 nm Ir(ppy)_2_(acac) doped Bepp_2_, 10 nm Bepp_2_, 50 nm Bphen ETL doped with LiF. Then, the transparent OLED was obtained by transferring TPHP(LiClO_4_)-coated graphene on the top of ETL using the same procedure for the fabrication of transparent QD-LED. The active area defined by the cathode of both LEDs was 4 × 4 mm^2^. The performances of devices were characterized same as those for transparent QD-LED.

#### OSCs

For the fabrication of OSCs, TPHP(CuCl_2_) polymer electrolyte was synthesized and spin-coated on graphene/PET substrates using the same synthesis and coating conditions as TPHP(LiClO_4_). To achieve better contact between the electrodes and the testing pins, and then reduce the resistance caused by the measurements, two Ag bus bars were thermally deposited on the edges of the TPHP(CuCl_2_)-coated graphene film, followed by deposition of a 30 nm MoO_x_ film. After that, a 300 nm photoactive layer comprising of poly[(2,5-bis(2-hexyldecyloxy)phenylene)-*alt*-(5,6-difluoro-4,7-di(thiophen-2-yl)benzo[c]-[1,2,5]thiadiazole)]:[6,6]-phenyl-C_71_-butyric acid methyl ester (PPDT2FBT:PC71BM) was deposited on the top of MoO_x_ by spin coating from a solution with a concentration of 35 mg mL^−1^ (1:1.5) in chlorobenzene containing 3 vol.% of diphenyl ether. Finally, we transferred the samples into a thermal evaporator to deposit 20 nm of Calcium (Ca) and 100 nm of Aluminum (Al) through a mask to define the device area as 0.2 cm^2^. The current-voltage curves were measured under carefully controlled conditions (in a glovebox with O_2_ < 1 ppm and H_2_O < 1 ppm) with a Keithley 2400 source-measurement unit under 1 sun illumination (AM 1.5 G, ~100 mW cm^−^^2^) with a 4-wire configuration and a scan rate of 200 mV s^−1^. The illumination intensity was 100 mW cm^−2^ from the solar simulator (ORIEL® Sol3A™ CLASS AAA SOLAR SIMULATOR) which was calibrated against a National Renewable Energy Laboratory (NREL) certified standard 2 cm × 2 cm silicon photodiode with no filter. The cells were excited through a 0.2 cm^2^ diameter mask that matched the size of the cells. The mismatch factor between the certified unfiltered silicon photodiode standard and the organic solar cells was determined to be 1.05. The reported power conversion efficiencies (PCEs) are corrected for the mismatch factor. External quantum efficiency (EQE) spectra were recorded with a PV Measurement QEX7 setup, which was operated without white light bias and chopped and locked in the small perturbation limit. Multiple devices (4–6) were fabricated and tested to establish statistically meaningful performance characteristics. Care was taken to ensure that in all cases the integrated EQE agreed with the white light short circuit current to within 10%.

#### PSCs

Patterned ITO substrates were ultrasonically cleaned with 2% Hellmanex water solution, deionized water, acetone, IPA and further treated by UV/ozone for 20 min. After depositing 20-nm-thick 2.2 eV TPHP(LiClO_4_), SnO_2_ solution (2.67%, diluted by water) was spin-coated at 3000 rpm for 25 s and annealed at 150 °C for 30 min under ambient conditions. Then, the perovskite precursor solution (1.12 mmol FAI, 0.21 mmol MABr, 0.85 mmol PbI_2_, 0.64 mmol PbBr_2_ and 47 μL CsI (1.5 M in DMSO) in 800 μL DMF and 200 μL DMSO mixed solvent) was spin-coated onto SnO_2_ at 4000 rpm for 40 s in glovebox. During the spin-coating process, 200 μL of chlorobenzene was dripped on the spinning substrate slowly 15 s prior to the end of the program. Then, the as-fabricated film was annealed at 120 °C for 30 min. After cooling down to room temperature, organic cation OABr (3 mg mL^−1^ in IPA) was spin-coated on perovskite films at 5000 rpm for 30 s and then annealed at 105 °C for 10 min to form 2D perovskite. Next, TPHP(CuCl_2_) was spin-coated at 5000 rpm for 45 s and heated at 120 °C for 10 min. After that, spiro-OMeTAD solution (73.2 mg mL^−1^ chlorobenzene, doped by 18 μL Li-TFSI, 29 μL FK209 Co(III) TFSI and 29 μL tBP) was spin-coated at 4000 rpm for 30 s and heated at 30 °C for 10 min. Finally, 80-nm-thick gold was thermally evaporated under a pressure of 10^−4 ^Pa as the back electrode.

The photovoltaic performances were measured under AM 1.5 G one sun illumination (100 mW cm^−2^) with a solar simulator (Newport 94021 A) calibrated by a Si-reference cell certified by NREL. The *J-V* curves of PSCs were recorded from 1.35 to −0.1 V with a linear scan step of 10 mV. The devices were covered with a metal mask to set the active area to 0.097 cm^2^.

### Statistical analysis

After removing a few abnormal data, data was presented using sample size (*n*), average value, standard deviation, and the parameters of Shapiro-Wilk tests (*p* and *α*), which were obtained by Origin software and Shapiro-Wilk test for normality evaluation. *p* > *α* represents normal distribution^56^.

In Fig. 2f, the error bars represent 10 samples’ (*n* = 10) standard error of the *WF* and electrical conductivities of TPHP and TPHP(LiClO_4_) with different mole ratio, respectively. The average value, standard deviation, *p* and *α* values of *WF* and electrical conductivity of TPHP and TPHP(LiClO_4_) with different mole ratio were shown in Supplementary Table 1 and 2. In Supplementary Fig. 25, 10 QD-LED devices with TPHP(LiClO_4_) EIL were measured (*n* = 10), showing that *L*_MAX_, *CE*_MAX_, and *PE*_MAX_ are 10663.7 ± 1870.16 cd m^−2^ (*p* = 0.235, *α* = 0.05), 11.96 ± 0.43 cd A^−1^ (*p* = 0.118, *α* = 0.05), and 6.12 ± 0.16 lm W^−1^ (*p* = 0.0535, *α* = 0.05), respectively. Note that *p* > *α* for all the performances.

### Reporting summary

Further information on research design is available in the Nature Research Reporting Summary linked to this article.

## Supplementary information

Supplementary Information

Description of Additional Supplementary Files

Supplementary Movie 1

Supplementary Movie 2

Reporting Summary

Solar Cells Reporting Summary

## Data Availability

The authors declare that the experimental data supporting the results of this study can be found in the paper and its Supplementary Information file. The detailed data for the study is available from the corresponding author upon request.
