# Supplementary material for: A polymer electrolyte design enables ultralow-work-function electrode for high-performance optoelectronics
Source: Nat Commun. 2022 Aug 25;13:4987. doi: 10.1038/s41467-022-32651-z (PMC9411633; doi:10.1038/s41467-022-32651-z)
Supplement: Supplementary file 1 — Supplementary Information [file 41467_2022_32651_MOESM1_ESM.pdf]

Supplementary Information for

A Polymer Electrolyte Design Enables Ultralow-Work-Function Electrode for High-Performance Optoelectronics

Tong *et al.*

Supplementary Figures:

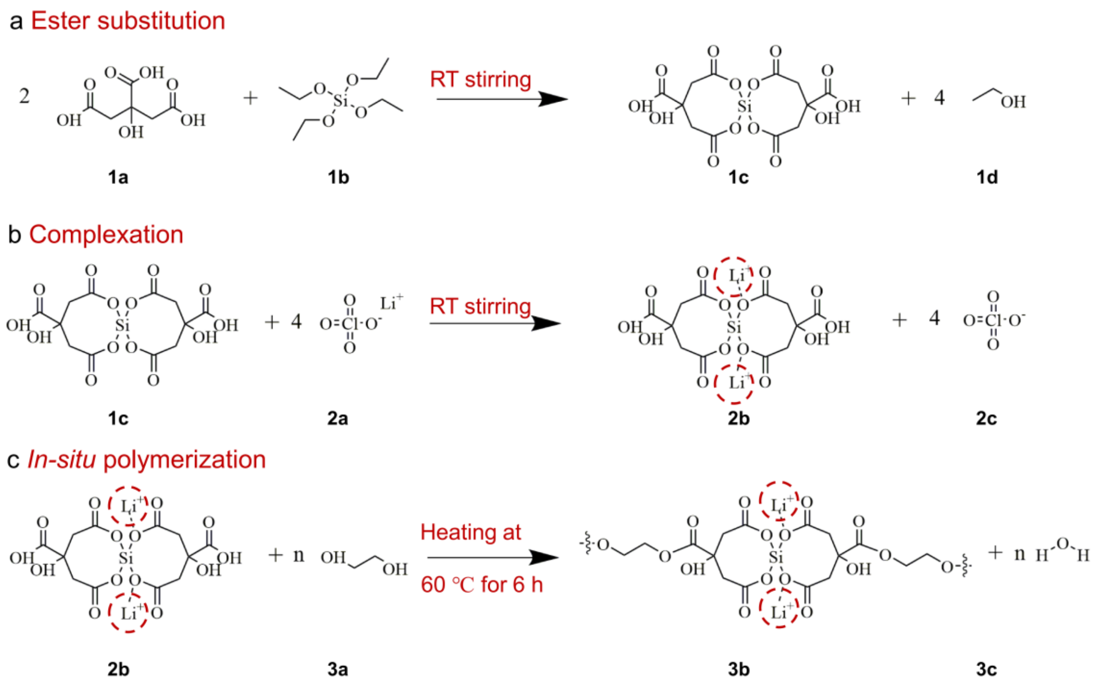

Supplementary Figure 1. The schematic of synthesis process of TPHP($LiClO_4$) polymer electrolyte. a, The ester substitution reaction between CA(**1a**) and TEOS(**1b**) forms siloxane monomer (**1c**). **b,** The siloxane monomer (**1c**) complexes with Li^+ of $LiClO_4$ (**2a**). **c,** *In-situ* polymerization of siloxane monomer (**2b**) with ethylene glycol (**3a**) forms TPHP($LiClO_4$) polymer electrolyte (**3b**).

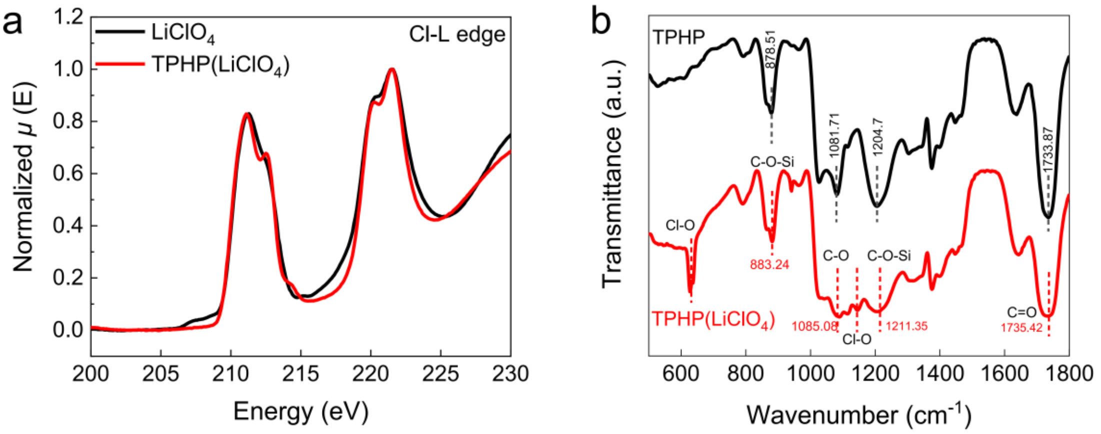

Supplementary Figure 2. The demonstration of reduction of ClO_4^- to low valence ClO_3^-. a, Full spectra of Cl L-edge XANES of $LiClO_4$ and TPHP($LiClO_4$). The mole ratio of Li^+/TPHP is 2:1. **b,** FT-IR absorption spectra of TPHP and TPHP($LiClO_4$). The mole ratio of Li^+/TPHP is 2:1.

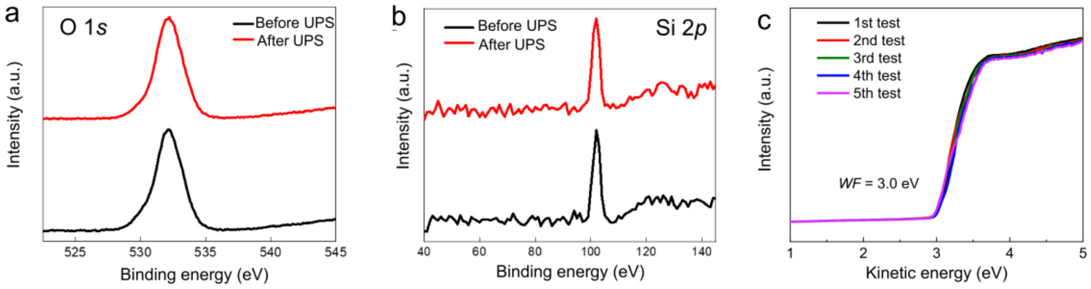

Supplementary Figure 3. Stability of TPHP($LiClO_4$) during UPS measurements. a, b, O 1*s* (**a**) and Si 2*p* (**b**) XPS spectra of TPHP($LiClO_4$) before and after UPS measurements. **c,** Multiple UPS tests on a TPHP($LiClO_4$) sample. The Li^+/TPHP mole ratio is 2:1 for all the measured samples.

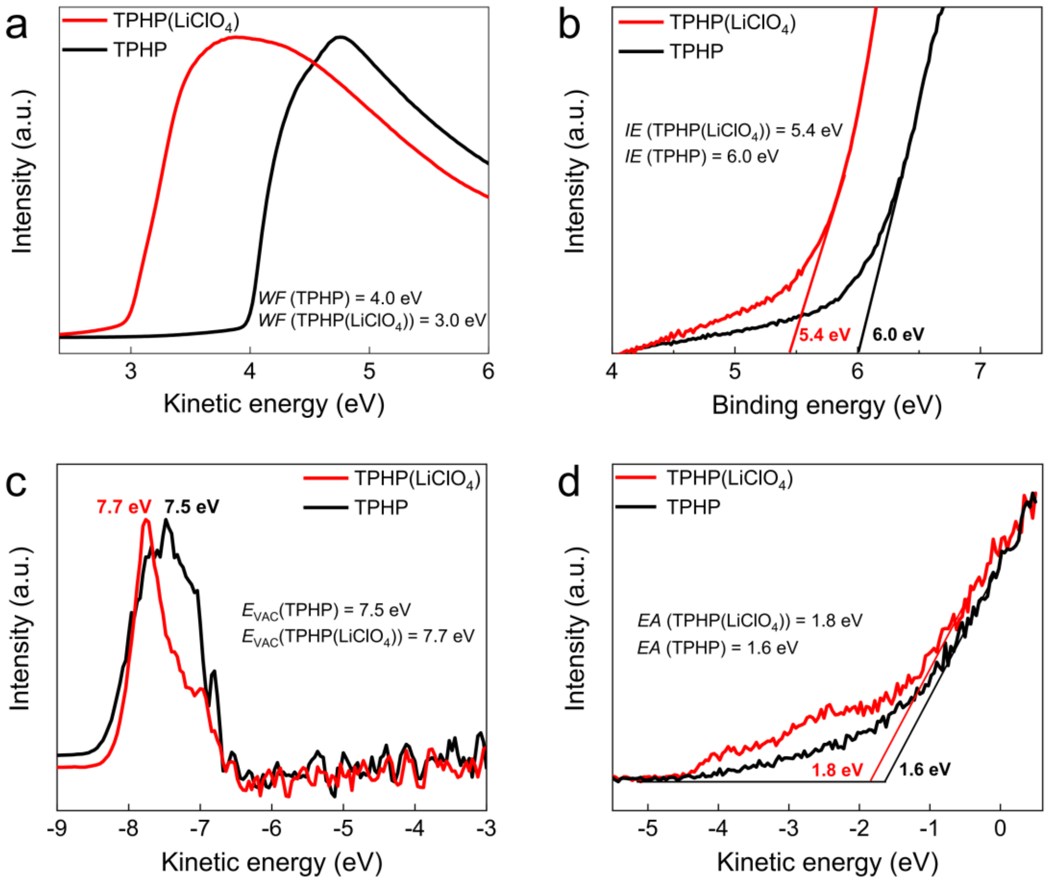

Supplementary Figure 4. UPS and IPES spectra of TPHP and TPHP($LiClO_4$). a, b, The low-energy cut-off edge (**a**) and the valence-band edge (**b**) of UPS spectra. **c, d,** E_{VAC} (**c**) and *EA* (**d**) of IPES spectra. The Li^+/TPHP mole ratio in TPHP($LiClO_4$) is 2:1.

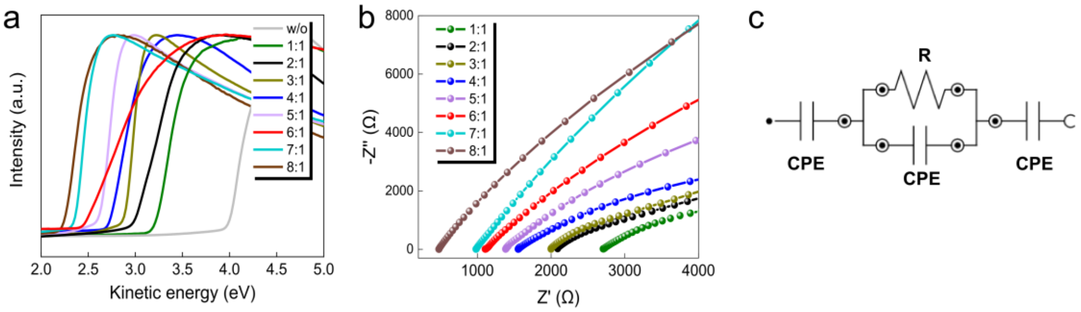

Supplementary Figure 5. Dependence of *WF* and electrical conductivity on the mole ratio of Li^+/TPHP. a, UPS spectra of TPHP and TPHP($LiClO_4$) with different mole ratio of Li^+/TPHP coatings on the top of Au. **b,** Nyquist plots of impedance for free-standing TPHP($LiClO_4$) film with different mole ratio of Li^+/TPHP. **c,** The equivalent circuit used to analyze the impedance data. R corresponds to the transport resistance of TPHP($LiClO_4$), and CPE corresponds to the dielectric capacitance between the film and electrode. The electrical conductivity σ of TPHP($LiClO_4$) film was calculated according to $\sigma = \frac{1}{R} \times \frac{L}{S}$, where L and S are the thickness and area, respectively.

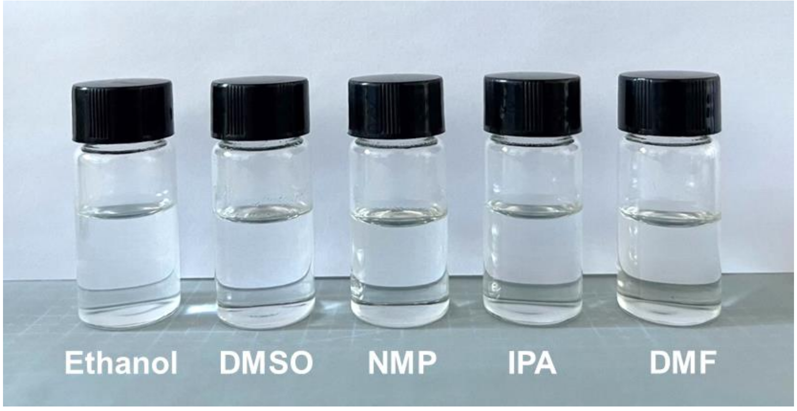

Supplementary Figure 6. Solubility of TPHP($LiClO_4$) in ethanol, DMSO, NMP, IPA and DMF.

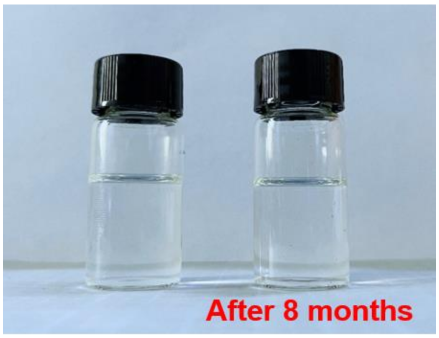

Supplementary Figure 7. Photograph of TPHP($LiClO_4$)/ethanol solutions that were just synthesized and have been stored under ambient conditions for 8 months.

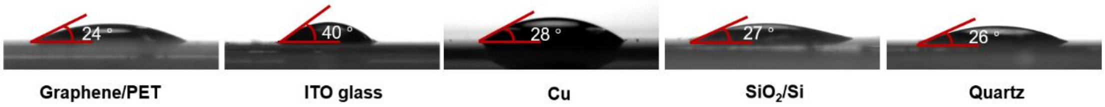

Supplementary Figure 8. Contact angles of TPHP($LiClO_4$)/ethanol solution on graphene/PET, ITO/glass, Cu, SiO_2/Si, and quartz substrates, showing good wettability.

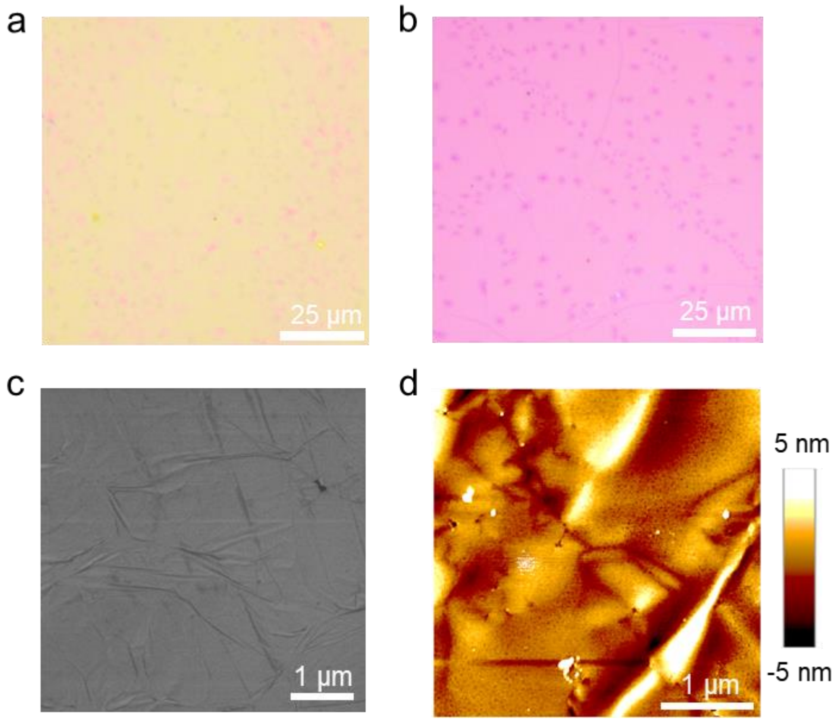

Supplementary Figure 9. a, b, OM images of transferred CVD-grown graphene films with (**a**) and without (**b**) TPHP($LiClO_4$) coating, which were placed on SiO_2/Si substrates. **c, d,** HIM (**c**) and AFM (**d**) images of CVD-grown graphene films without TPHP($LiClO_4$) coating, which were placed on TRT substrates.

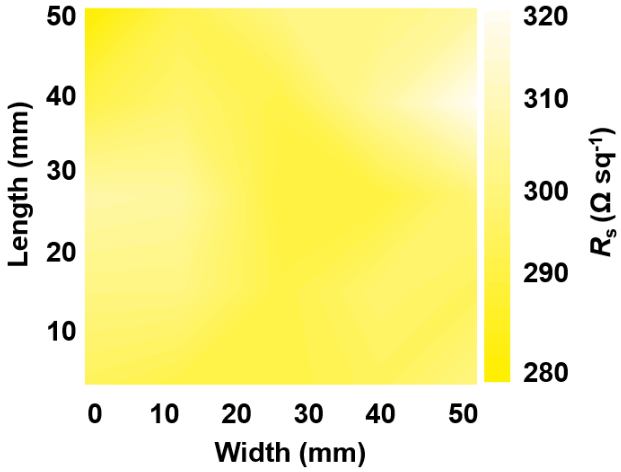

Supplementary Figure 10. R_s mapping of the transferred CVD-grown graphene film with TPHP($LiClO_4$) coating, which was placed on PET substrate.

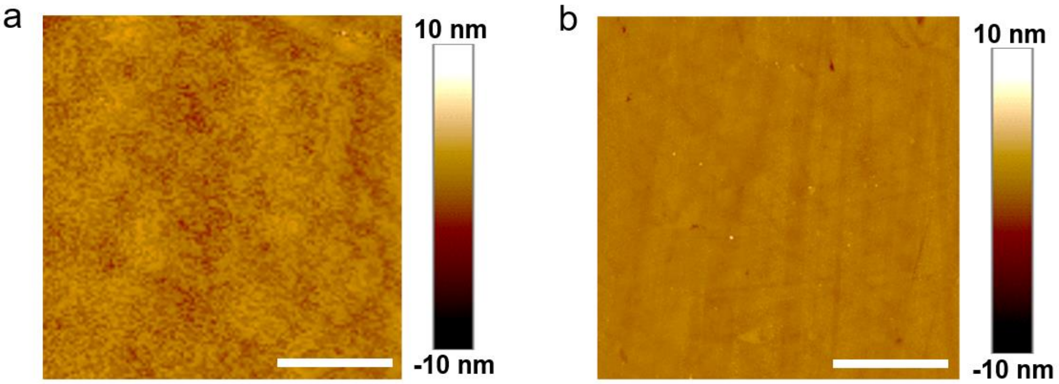

Supplementary Figure 11. AFM images of TPHP($LiClO_4$)-coated fused silica. TPHP($LiClO_4$) was deposited from ethanol (**a**) and NMP (**b**) solution. The scale bars are 1 μm.

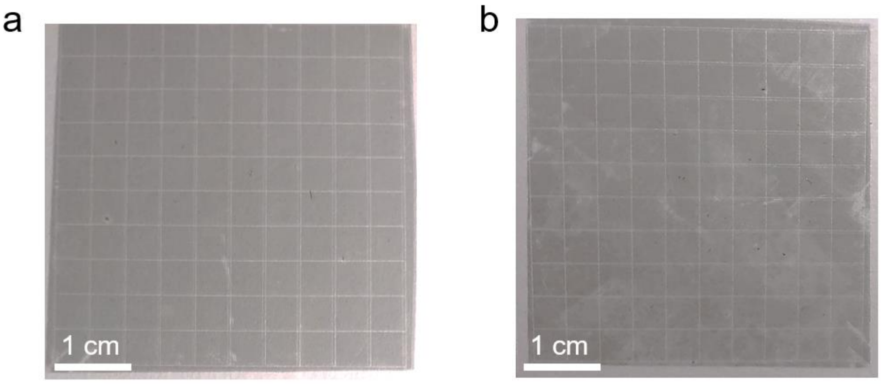

Supplementary Figure 12. TPHP($LiClO_4$)-coated CVD-grown graphene transferred from TRT to ZnO/glass (**a**) and after being peeled off by blue film tape (**b**). Most of the squares (0.25×0.25 mm^2 each) are intact and the edges are quite sharp after transferring from TRT to ZnO/glass. Further peeling by blue film tape still can keep the sharp edges of most of graphene squares and only a small fraction was peeled out, showing the good binding capability of TPHP($LiClO_4$) coating.

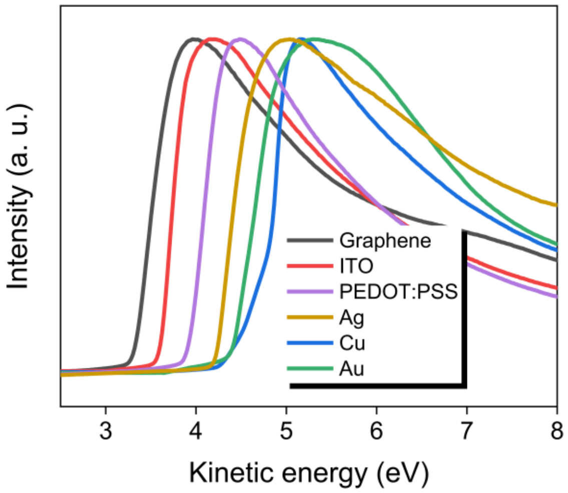

Supplementary Figure 13. Photoemission cutoff obtained via UPS for graphene, ITO, PEDOT:PSS PH1000, Au, Ag and Cu conductors after coating 3.0 eV TPHP($LiClO_4$).

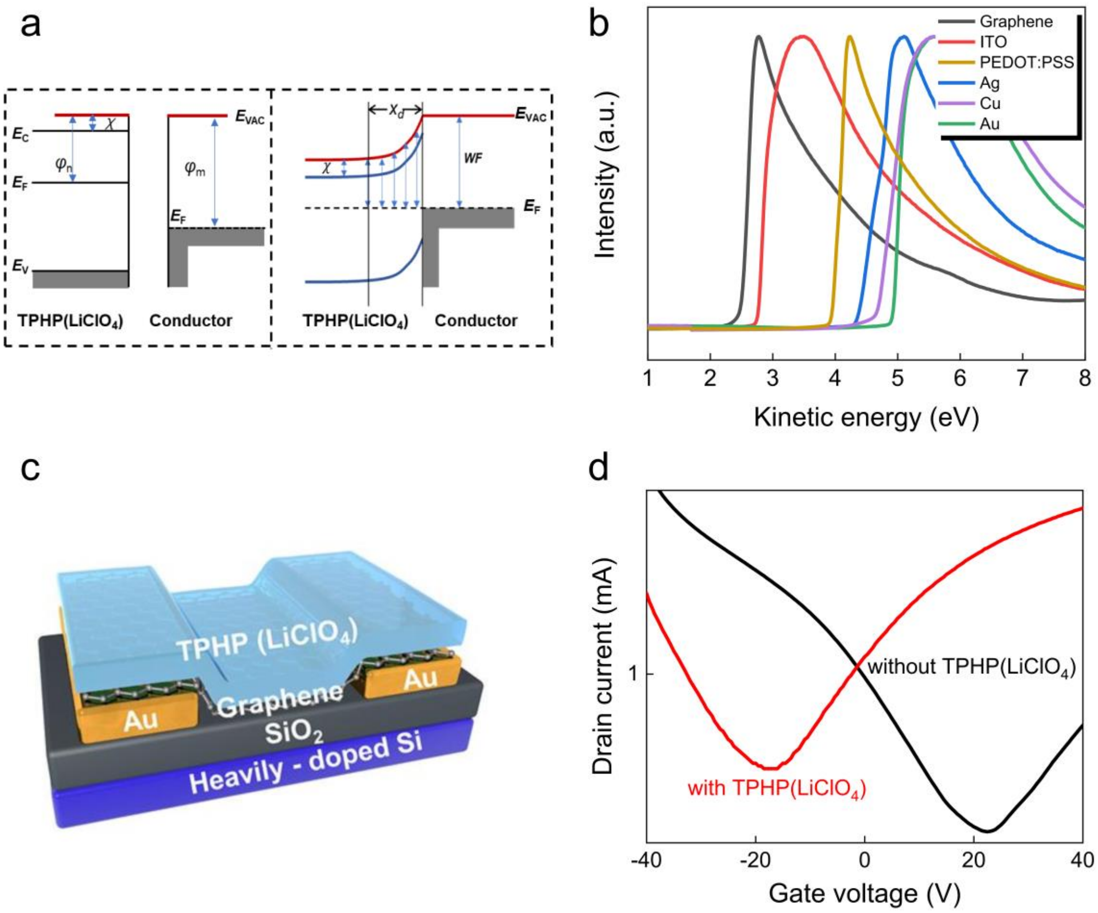

Supplementary Figure 14. The *WF* modification mechanism of TPHP($LiClO_4$) on conductors. a, Energy band diagram of TPHP($LiClO_4$) and a conductor before (left) and after contact with each other (right), in which E_{VAC} is the vacuum energy level, E_C is the conduction band, E_V is the valence band, E_F is Fermi level, χ refers to electron affinity, X_d denotes the width of space charge region, φ_n and φ_m represent the *WF* of TPHP($LiClO_4$) and conductor, respectively. **b,** Photoemission cutoff obtained via UPS on the uncovered side of graphene, ITO, Ag, Au, Cu and PEDOT:PSS electrodes with the other half being covered with 30 nm-thick 2.2 eV TPHP($LiClO_4$). **c,** Schematic of the transistor with TPHP($LiClO_4$)-coated graphene channel. **d,** Transfer characteristics of the graphene transistors with and without TPHP ($LiClO_4$) coating.

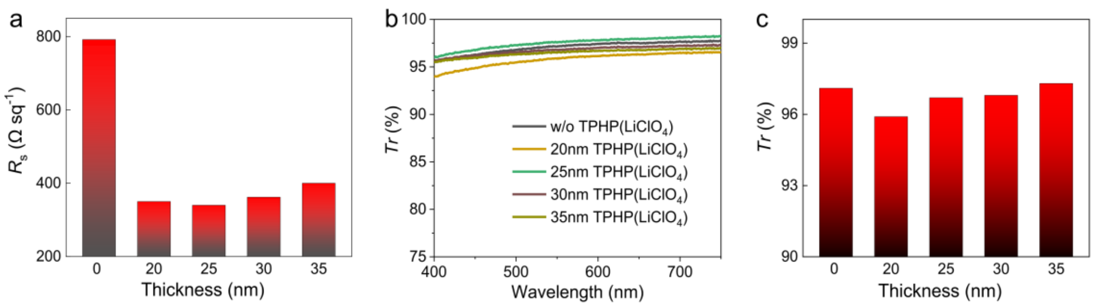

Supplementary Figure 15. a, R_s for graphene coated with different thickness of TPHP($LiClO_4$). **b,** *Tr* spectra of graphene coated with different thickness of TPHP($LiClO_4$) on quartz substrate. **c,** The corresponding *Tr*@550 nm wavelength for graphene coated with different thickness of TPHP($LiClO_4$).

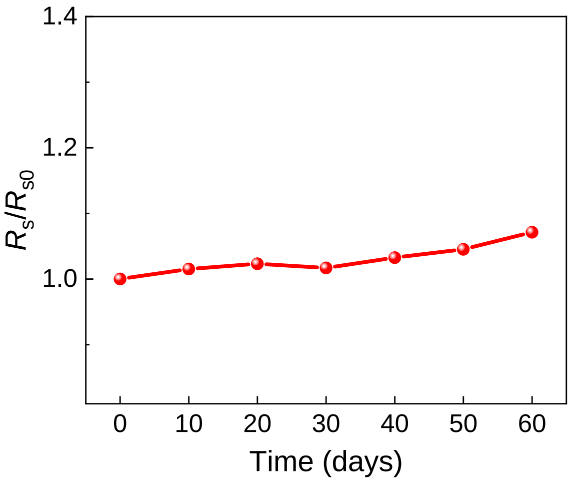

Supplementary Figure 16. R_s change of the TPHP($LiClO_4$)-coated graphene as a function of time.

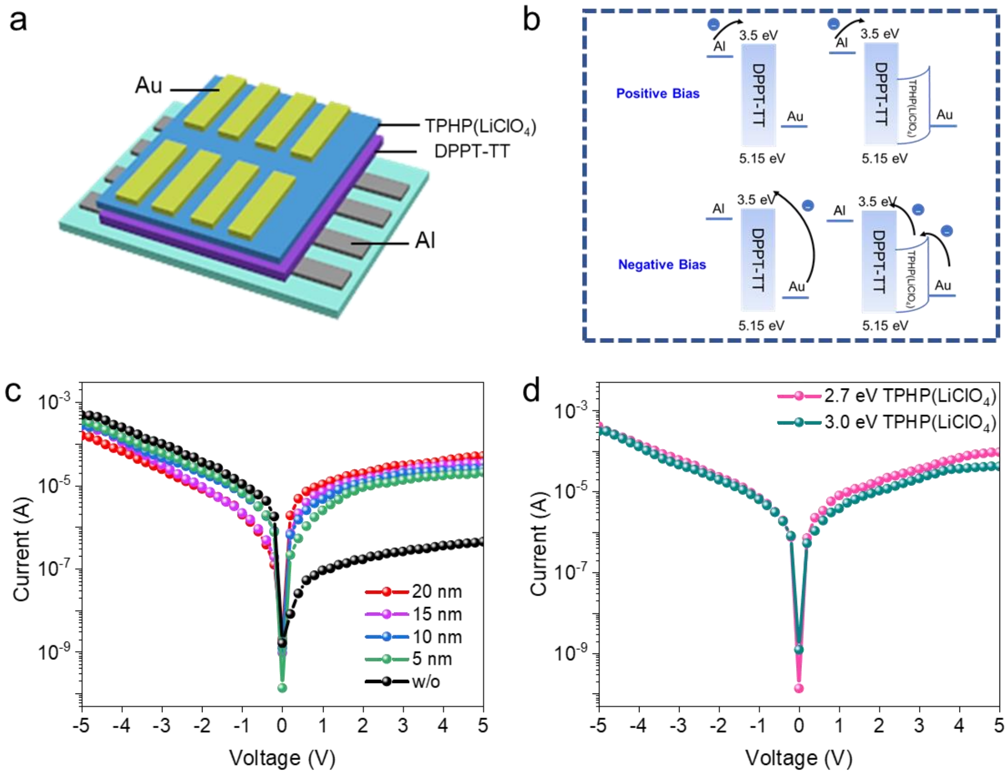

Supplementary Figure 17. Tuning the electrical characteristics of Schottky diode by TPHP($LiClO_4$). a, Schematic of Schottky diode using Al and Au as electrodes and DPPT-TT as n type semiconductor. **b,** Energy level diagrams. **c, d,** Typical *I-V* characteristics for devices with TPHP($LiClO_4$) of different thickness (**c**) and different *WF* (**d**). The *WF* of TPHP($LiClO_4$) is 2.2 eV in **c** and the thickness of TPHP($LiClO_4$) is 20 nm in **d**.

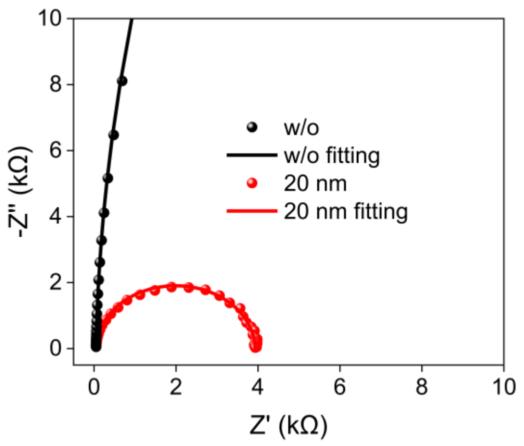

Supplementary Figure 18. Cole-Cole plots biased at 1 V for blue QD-LEDs without and with 20 nm-thick 2.2 eV TPHP($LiClO_4$) EIL, showing the significant reduction of the interface resistance between Ag and blue QDs with the use of TPHP($LiClO_4$) EIL.

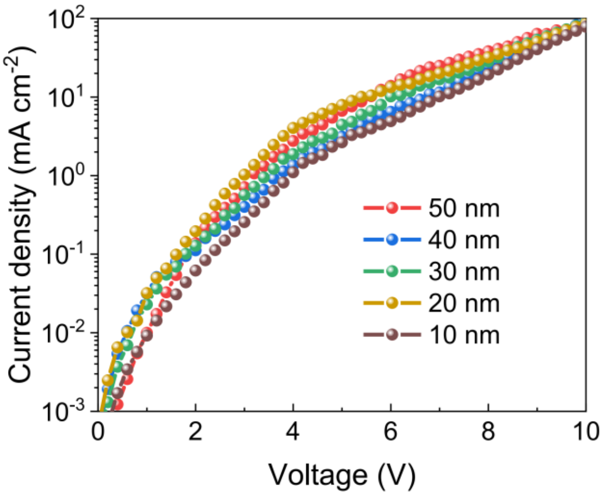

Supplementary Figure 19. *J-V* characteristics for the devices with different thickness of 2.2 eV TPHP($LiClO_4$) EIL.

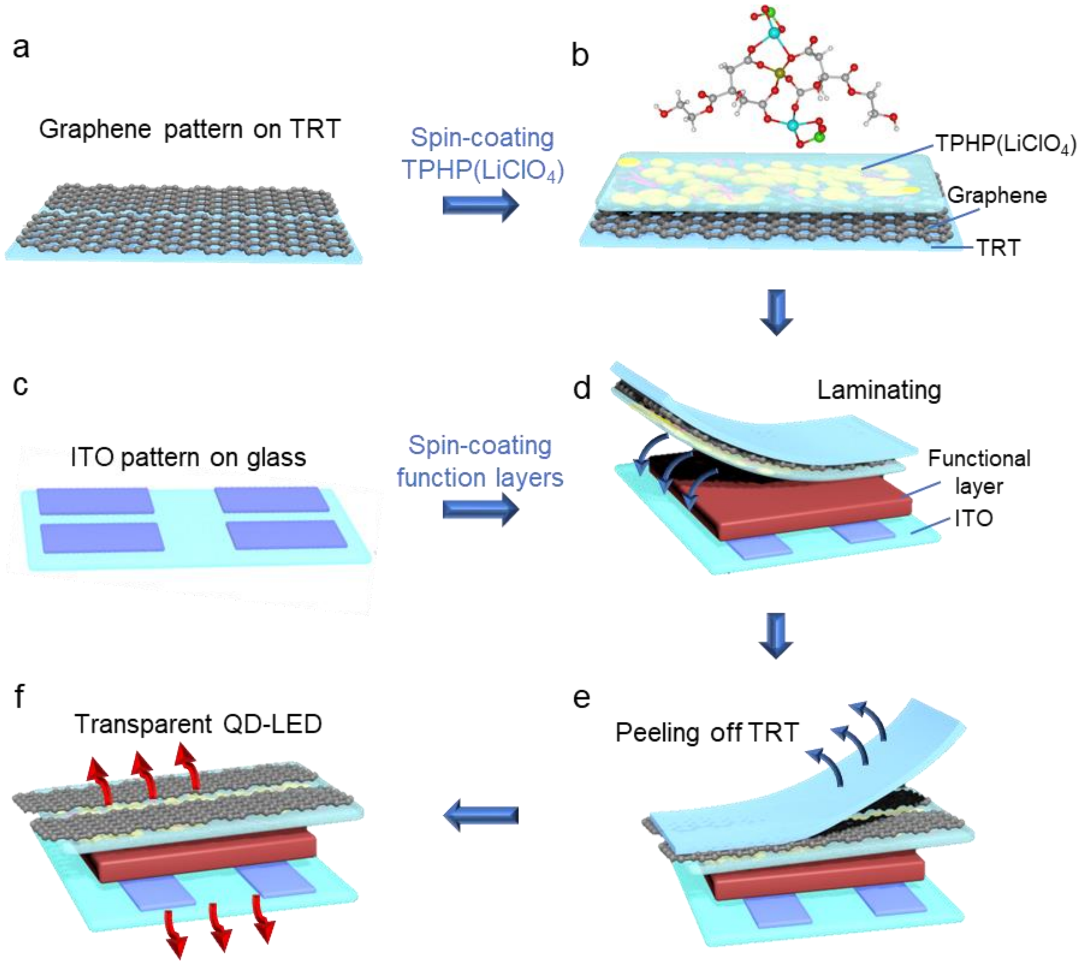

Supplementary Figure 20. Schematic of the fabrication process of transparent QD-LED with graphene top electrode and TPHP($LiClO_4$) EIL. a, 3L graphene on TRT. **b,** The TPHP($LiClO_4$)-coated graphene on TRT obtained by spin-coating TPHP($LiClO_4$) on graphene. **c,** ITO pattern on glass. **d,** ITO/PEDOT:PSS/PVK/ ZnCdSe/ZnSeS/ZnCdS QDs light emitting layer/ZnO QD ETL/TPHP($LiClO_4$) EIL/graphene/TRT stack obtained by depositing PEDOT:PSS, PVK, ZnCdSe/ZnSeS/ZnCdS and ZnO QDs on ITO in sequence and then laminating graphene/TRT on the top of ZnO ETL. **e,** Peeling off TRT from graphene. **f,** A transparent QD-LED with pure graphene as top cathode.

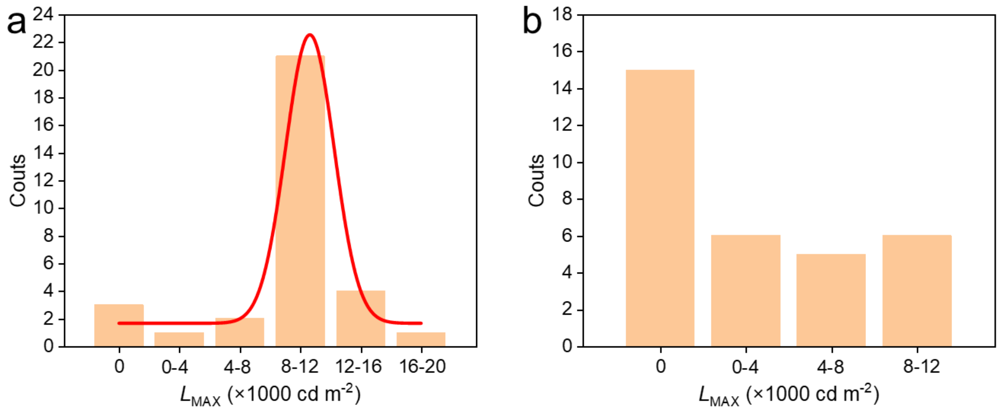

Supplementary Figure 21. L_{MAX} distribution of 32 QD-LED devices with (**a**) and without (**b**) TPHP($LiClO_4$) EIL.

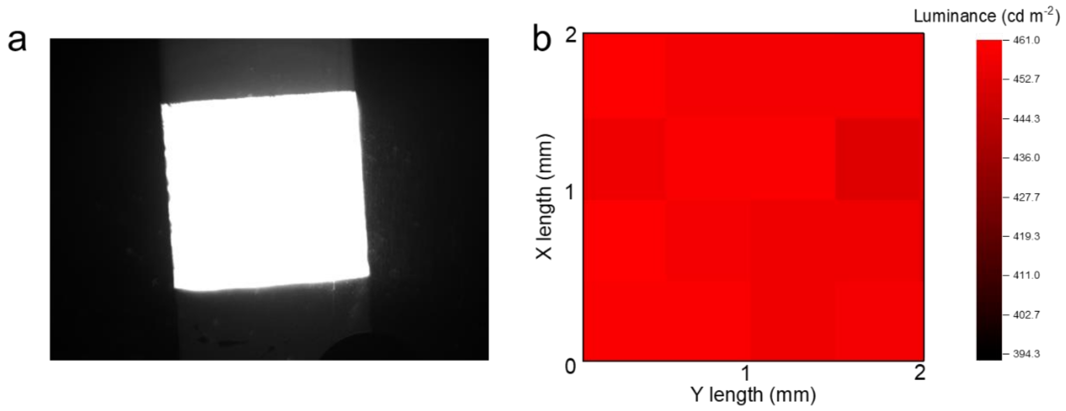

Supplementary Figure 22. Homogeneity of the device with TPHP($LiClO_4$) EIL under operation. a, Dark field optical image of a lighted QD-LED. **b,** EL spectra mapping.

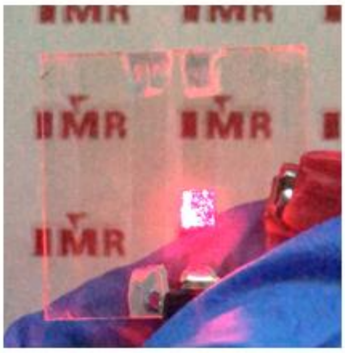

Supplementary Figure 23. A lighted QD-LED using 3L pure graphene as top cathode without TPHP($LiClO_4$) EIL.

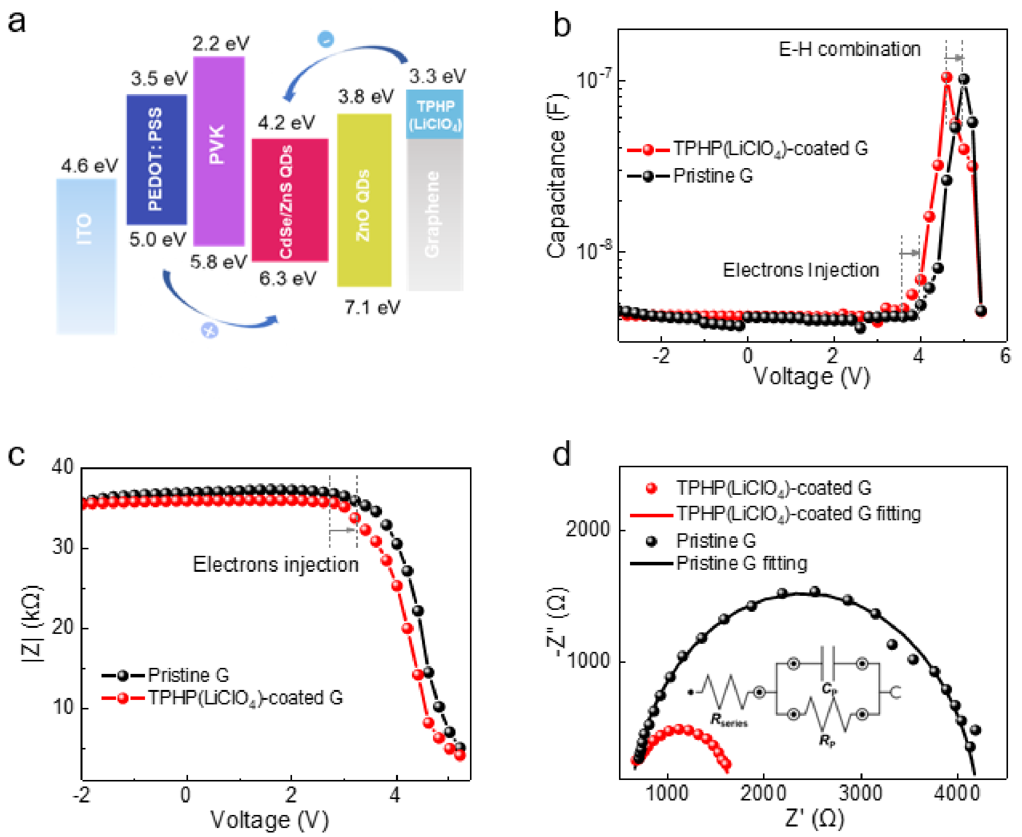

Supplementary Figure 24. Energy level diagram and impedance spectroscopy characterization of transparent QD-LEDs with graphene (G) top electrode. a, Energy level diagram of QD-LED with TPHP($LiClO_4$) EIL. **b, c, d,** *C*-*V* (**b**) and *Z*-*V* (**c**) characteristics at the frequency of 1000 Hz and Cole-Cole plots biased at 4 V (**d**) for QD-LED with and without TPHP($LiClO_4$) EIL. The inset in **d** is the equivalent circuit, in which R_{series} means the resistance of electrode, R_p means the interfacial contact resistance and C_p means the interface capacitance.

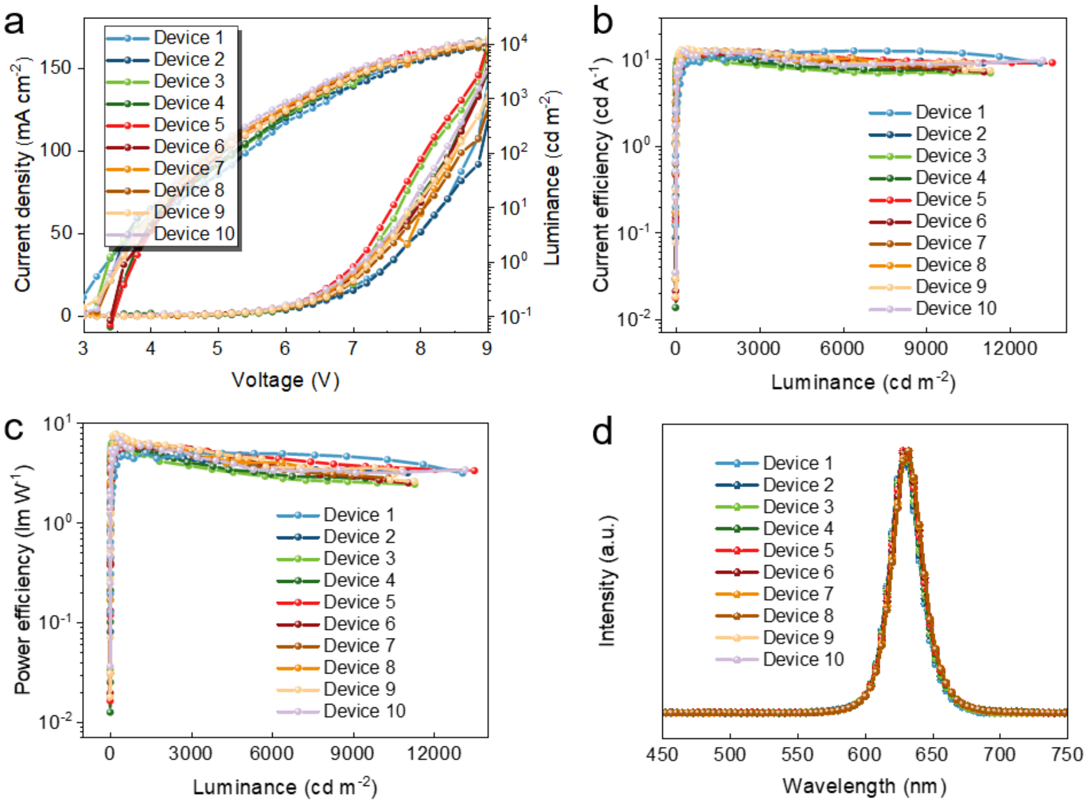

Supplementary Figure 25. Performances of 10 QD-LEDs with TPHP($LiClO_4$) EIL. **a,** *J-V-L* characteristics. **b,** *CE-L* curves. **c,** *PE-L* curves. **d,** EL spectra. It can be found that our devices achieve maximum luminance of ~13,000 cd m^{-2} at 9 V. Further increasing voltage leads to luminance degradation and the ablation of devices, because the excess Joule heat generated at higher voltage induces emission quenching of QDs like most reported QD-LEDs. Actually, most LEDs for electronic devices, such as smart phone, generally work below ~6 V with a luminance of ~100 cd m^{-2}. Therefore, our devices should be sufficient for display applications in terms of operation voltage and luminance. The experimental errors of the transparent QD-LED performances are mainly due to the top electrode transfer process and the small difference in each layer thickness of QD-LED arising from the spin-coating process.

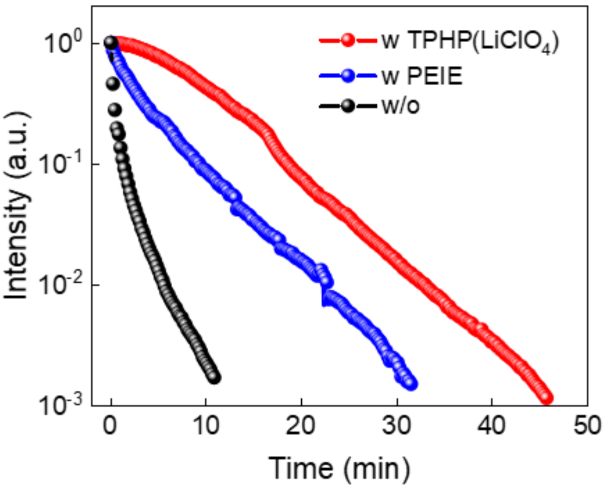

Supplementary Figure 26. Normalized luminance of the QD-LEDs with different EIL as a function of operating time at a constant current density and an initial luminance of ~1000 cd m^{-2}.

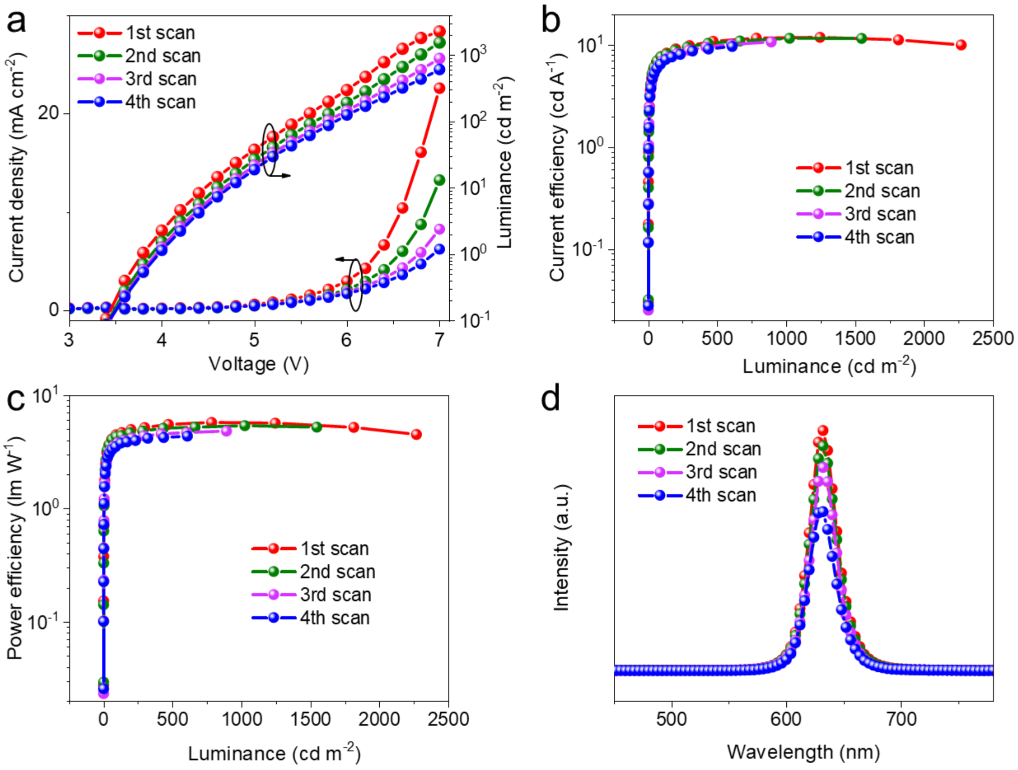

Supplementary Figure 27. Device performances after running 4 times from 0 to 7 V. a, *J-V-L* characteristics. **b,** *CE-L* and **c,** *PE-L* curves. **d,** EL spectra.

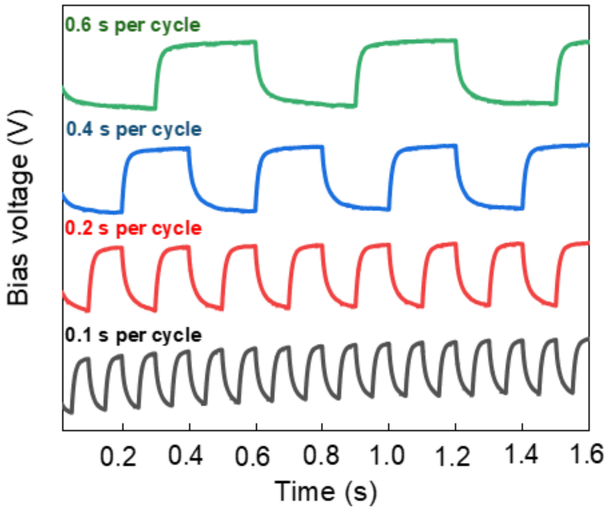

Supplementary Figure 28. Transient effects characterization of QD-LEDs with TPHP($LiClO_4$) EIL. Charging and discharging tests of QD-LEDs were performed with different sweep rates (0.1 s per cycle, 0.2 s per cycle, 0.4 s per cycle and 0.6 s per cycle) under a constant current of 0.1 mA. The obtained turn-on time and turn-off time are 0.20 ms and 0.45 ms, respectively, both of which are very fast. Furthermore, they do not change with sweeping rate and turn on times, suggesting that there are no transient effect during device operation.

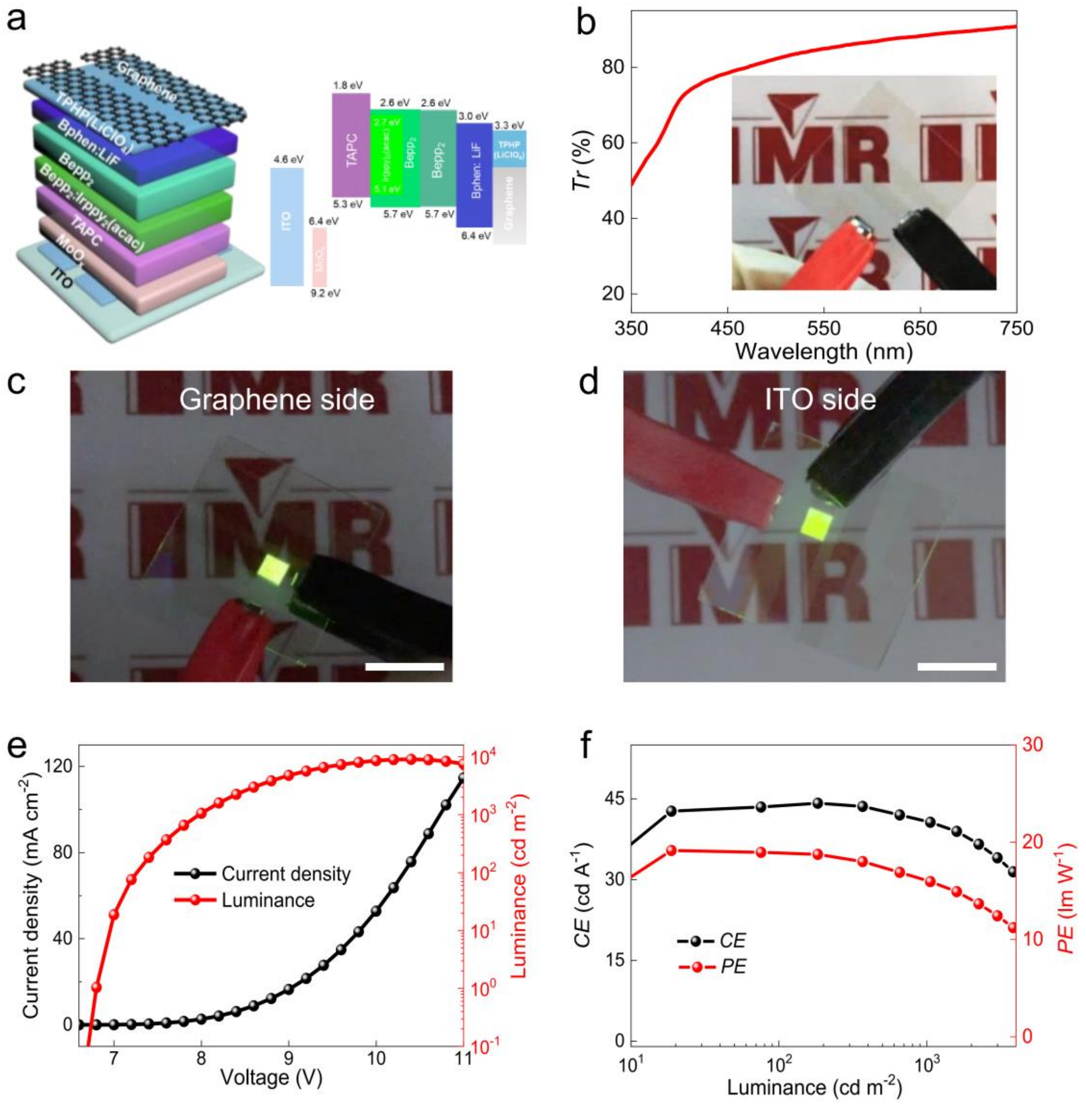

Supplementary Figure 29. Structure and performance of transparent green OLED with graphene top electrode and TPHP($LiClO_4$) EIL. a, Device structure (left) and energy level diagram (right). **b,** *Tr* spectrum of the device (inset). **c, d,** Photographs of the lighted OLED from graphene (**c**) and ITO (**d**) sides. **e, f,** *J*-*V*-*L* (**e**) and *CE*- and *PE*-*L* (**f**) characteristics. Scale bars in **c** and **d** are 1 cm.

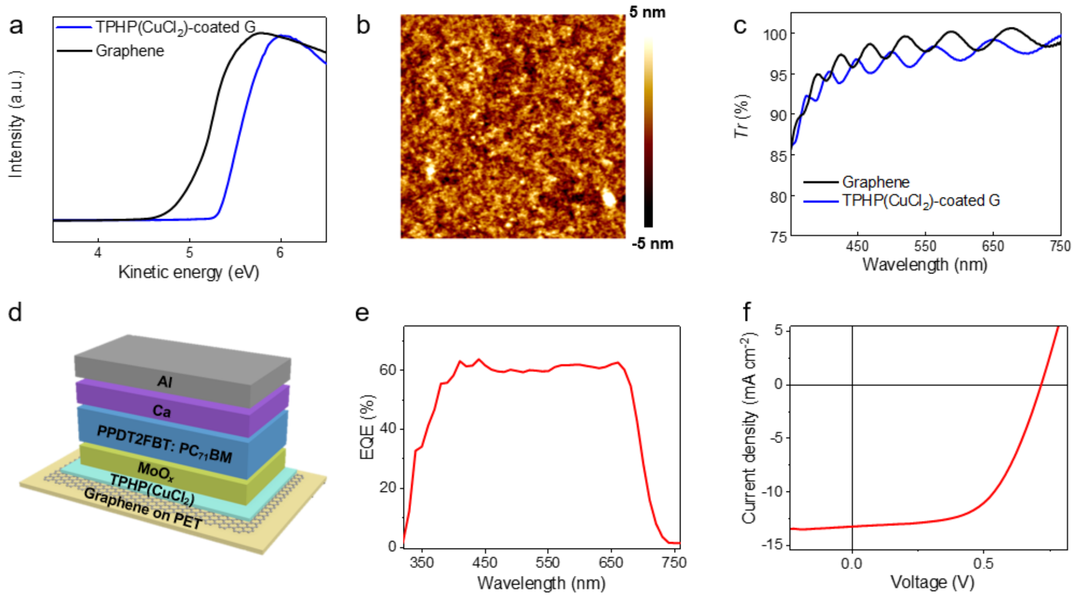

Supplementary Figure 30. Structure and properties of TPHP($CuCl_2$)-coated graphene. a, UPS spectra of graphene and TPHP($CuCl_2$)-coated graphene (G). **b,** AFM image of TPHP($CuCl_2$)-coated graphene film on PET substrate. **c,** *Tr* spectra of graphene on PET substrate before and after TPHP($CuCl_2$) coating. **d,** Device structure of OSC with graphene bottom electrode and TPHP($CuCl_2$) HIL. **e,** External Quantum efficiency (EQE). **f,** *J*-*V* characteristic showing the short-circuit current of 15.13 mA cm^{-2}.

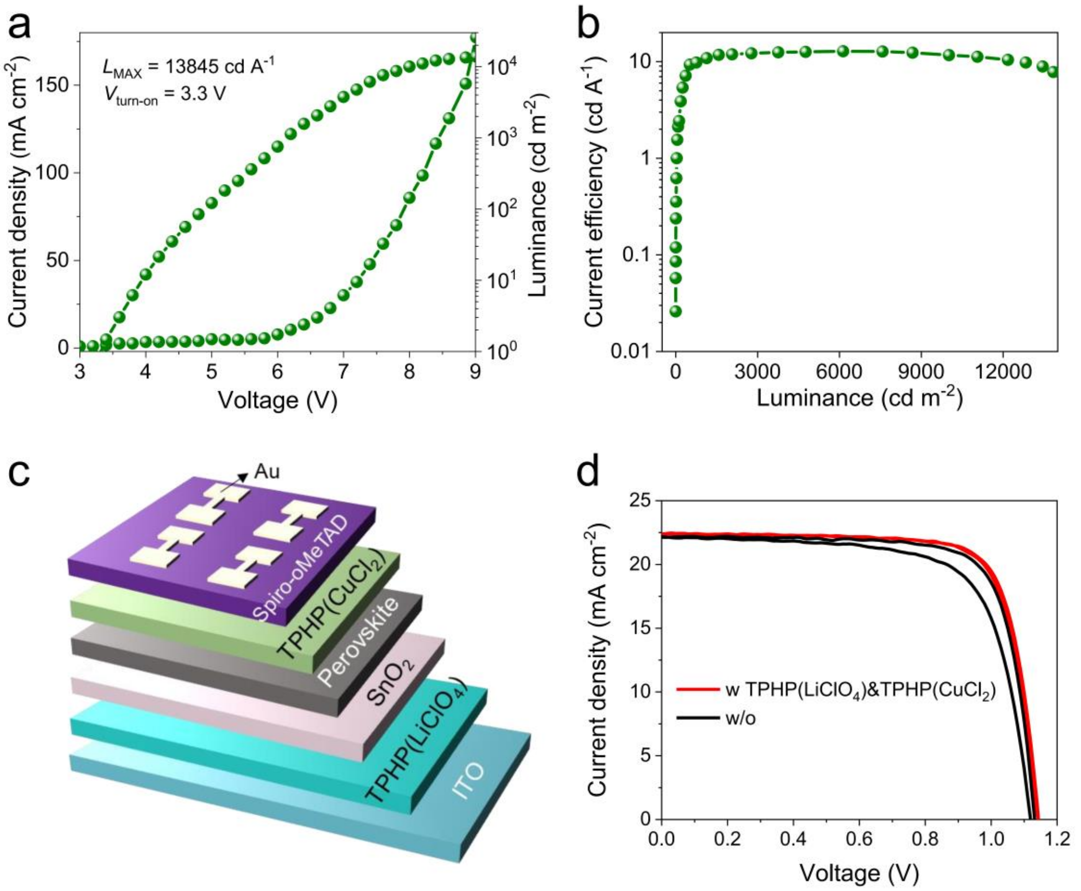

Supplementary Figure 31. Performances of transparent QD-LEDs and PSCs. a, b *J*-*V*-*L* (**a**) and *CE*-*L* (**b**) characteristics of the transparent QD-LED with TPHP ($CuCl_2$) as HIL and TPHP($LiClO_4$) as EIL. **c,** Schematic device structure of PSCs. **d,** *J*-*V* curves of devices with/without TPHP($LiClO_4$) EIL and TPHP($CuCl_2$) HIL.

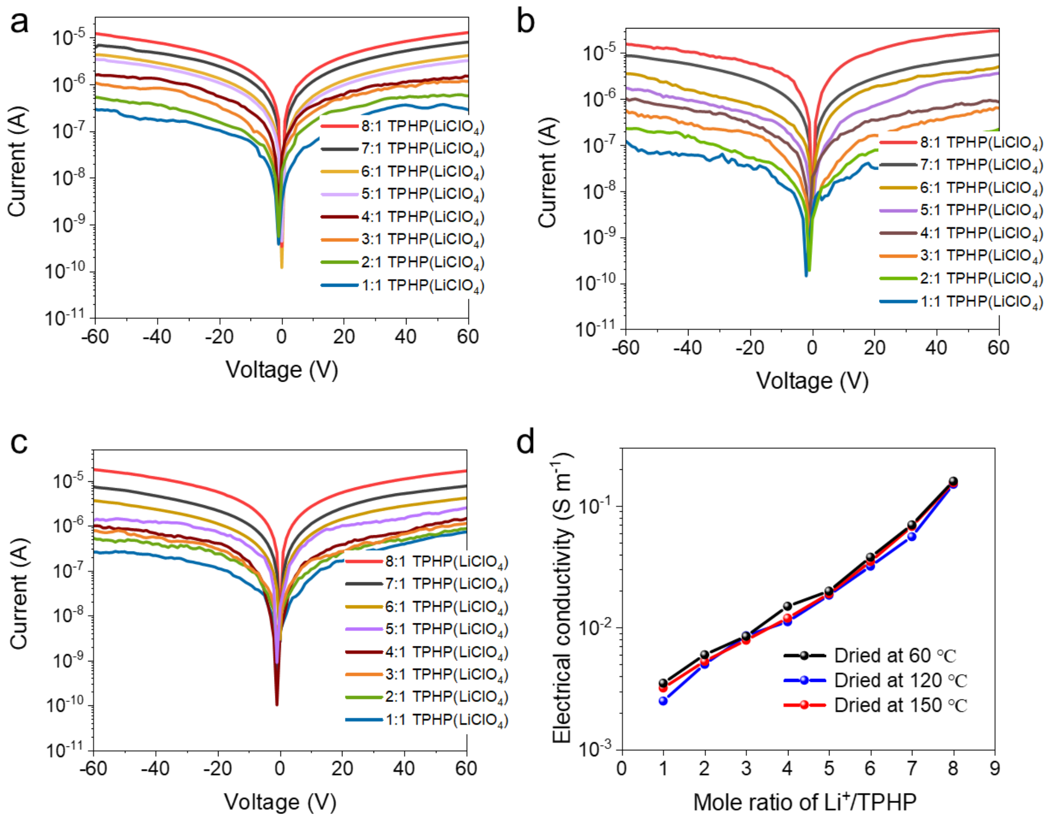

Supplementary Figure 32. a-c, *I-V* characteristics of TPHP($LiClO_4$) thin films with different mole ratio of Li^+/TPHP dried at 60 °C (**a**), 120 °C (**b**) and 150 °C (**c**), respectively. **d**, Dependence of electrical conductivity on the mole ratio of Li^+/TPHP for TPHP($LiClO_4$) dried at 60 °C, 120 °C and 150 °C, respectively. Almost the same conductivity indicates that drying at 60 °C for 2 h is enough to obtain dried TPHP($LiClO_4$) to avoid the influence of hydrolyzed electrolyte on the conductivity. Moreover, the electrical conductivities obtained based on devices are consistent with those achieved based on impedance measurements, confirming the good electron transport properties of TPHP($LiClO_4$).

Supplementary Tables

Supplementary Table 1. The average value, standard deviation, *p* and α values of electrical conductivities of TPHP and TPHP($LiClO_4$) with different mole ratio of Li^+/TPHP.

	Average ($S\ m^{-1}$)	Standard deviation ($S\ m^{-1}$)	*p*	*α*
TPHP	8×10^{-10}	4×10^{-10}	0.0347	0.05
TPHP($LiClO_4$) 1:1	0.0234	0.020	0.557	0.05
TPHP($LiClO_4$) 2:1	0.0303	0.010	0.265	0.05
TPHP($LiClO_4$) 3:1	0.0321	0.015	0.326	0.05
TPHP($LiClO_4$) 4:1	0.0411	0.025	0.0369	0.05
TPHP($LiClO_4$) 5:1	0.0460	0.018	0.263	0.05
TPHP($LiClO_4$) 6:1	0.0573	0.012	0.237	0.05
TPHP($LiClO_4$) 7:1	0.0656	0.021	0.0269	0.05
TPHP($LiClO_4$) 8:1	0.137	0.005	0.266	0.05

* The experimental errors of electrical conductivity mainly originate from the contact resistance between electrode and TPHP($LiClO_4$).

Supplementary Table 2. The average value, standard deviation, *p* and α values of *WF* of TPHP and TPHP($LiClO_4$) with different mole ratio of Li^+/TPHP.

	Average (eV)	Standard deviation (eV)	p	α
TPHP	4.00	0.087	0.149	0.05
TPHP($LiClO_4$) 1:1	3.10	0.120	0.575	0.05
TPHP($LiClO_4$) 2:1	3.00	0.120	0.392	0.05
TPHP($LiClO_4$) 3:1	2.80	0.089	0.045	0.05
TPHP($LiClO_4$) 4:1	2.67	0.087	0.452	0.05
TPHP($LiClO_4$) 5:1	2.55	0.110	0.097	0.05
TPHP($LiClO_4$) 6:1	2.41	0.050	0.097	0.05
TPHP($LiClO_4$) 7:1	2.29	0.050	0.404	0.05
TPHP($LiClO_4$) 8:1	2.20	0.120	0.038	0.05

* The experimental errors of *WF* mainly arise from the states of materials including surface contamination and roughness [13].

Supplementary Table 3. *WF* measured by UPS for various conductors with half being coated with 30 nm-thick 2.2 eV TPHP($LiClO_4$). The *WF* reduction of the other half without TPHP($LiClO_4$) coating represents the E_F change of the conductor.

Material	Pristine (eV)	With TPHP($LiClO_4$) (eV)	Δ*WF* (eV)	Uncovered side (eV)	Δ*WF* (eV)
**Graphene**	4.65	2.20	2.45	2.40	2.25
**ITO**	4.50	2.52	1.98	2.66	1.84
**PEDOT:PSS**	4.95	3.60	1.35	3.86	1.09
**Au**	5.10	3.35	1.75	5.00	0.10
**Ag**	4.62	3.51	1.11	4.42	0.20
**Cu**	4.60	3.31	1.29	4.56	0.04

Supplementary Table 4. Summary of *WF*, R_s and *Tr*@550 nm wavelength of graphene coated with different thickness of TPHP($LiClO_4$) EIL.

**Thickness (nm)**	**0 (Pristine)**	**20.0**	**25.0**	**30.0**	**35.0**
R_s **(Ω·sq^{-1})**	800	350	345	355	460
Tr **(%@550 nm wavelength)**	97.1	95.9	96.7	96.8	97.3
FoM[a]	16.06	25.46	34.94	31.76	34.20

[a]*FoM* (figure of merit) values were calculated by $Tr_{(\lambda)}=\left(1+\frac{188.5}{R_s \times FoM}\right)^{-2}$, where $Tr_{(\lambda)}$ is the *Tr* of graphene at 550 nm wavelength [1].

Supplementary Table 5. Device performances of transparent red QD-LEDs based on different top electrodes. All the devices were fabricated on glass substrates.

**Ref.**	**Top electrode**	**Preparation method of top electrode**	**Device structure**	***CE*$_{MAX}$ (cd A^{-1})**	***PE*$_{MAX}$ (lm W^{-1})**	***L*$_{MAX}$ (cd m^{-2})**	***Tr%@* 550 nm**
**[2]**	Ag	Evaporation	ITO/AZO: Cs_2CO_3/QDs/TCTA/NPD/ HAT-CN/Ag	1.25	0.67	10540 (at ~12 V)	~45
**[3]**	IZO	Sputtering	ITO/ZrO_2/InP QDs/TAPC /MoO_3/IZO	0.47	-	200 (at ~16 V)	>74
**[4]**	Ag NWs/ 8L Graphene	Roll-in	Au-NPs/graphene /PEDOT:PSS/PVK /CdZnSeS/ZnS QDs/ZnO NPs/Ag NW/graphene	0.45	-	~358 (at ~29 V)	70-80
**[5]**	Graphene/ PDMS/PET	Roll-in	ITO/ZnO/CdSSe /ZnS/TAPC/MoO_3 /G/PDMS	0.32	0.19	42 (at ~9.8 V)	78
**[6]**	Ag NWs	Roll-in	ITO/ZnO/QDs/PVK/PEDOT:PSS/ Ag NW_S	1.55	-	10011 (at ~15 V)	75
**Our Work**	**3L graphene**	**Roll-in**	**ITO/PEDOT:PSS /PVK/ ZnCdSe/ZnSeS/ZnCdS QDs/ZnO QDs/ TPHP ($LiClO_4$)/ Graphene**	**12.55**	**5.03**	**~13000 (at ~9.0 V)**	**85.1**

*For our transparent QD-LEDs, we used a simple device structure without special design, in which the functional layer materials are similar to those of the devices reported in Supplementary Ref. 4 and 6. It has been reported the performance of QD-LEDs is greatly dependent on the photoluminescence quantum yield (PLQY) of the QD active layer. We used commercial red QDs of ZnCdSe/ZnSeS/ZnCdS. The PLQY is only 61.2%, similar to or even lower than that of the light-emitting layer materials used in the literature. Therefore, the comparison results with those previously reported in literature give reliable evidence of the advantages of the use of TPHP($LiClO_4$) EIL in optoelectronic devices.

Supplementary Table 6. Parameters for simulating the Cole–Cole plots.

Device	R_{series} (Ω)	R_p (kΩ)	C_p (nF)
**With TPHP($LiClO_4$) EIL**	622	1.01	5.12
**With PEIE EIL**	745	2.20	4.23
**Without EIL**	708	3.22	2.86

Supplementary Table 7. Device performances of transparent green OLEDs based on different top electrodes.

Ref.	Top electrode	Preparation method of electrode	Device structure	CE_{MAX} (cd A^{-1})	L_{MAX} (cd m^{-2})	*Tr*%@ 550 nm	Substrate
**[7]**	ITO (90 nm)	Magnetron sputtering	AZO/Bphen:Li/ TPBI/TPBI:Ir(ppy)3/ TCTA:Ir(ppy)3/TAPC/ Pentacene/1-TNATA/ PEDOT:PSS/ITO	-	700 (at ~5 V)	68	Glass
**[8]**	Al/Ag	Evaporation	ITO/WO_3/NPB/Alq_3: DCM/LiF/Al/Ag/NPB	-	-	>45	Glass
**[9]**	Ag (25 nm)	Evaporation	ITO or PEDOT:PSS/Spiro-TAD/NPB:Ir(MDQ)2 (acac)/BAlq2/Bphen:Cs /Ag/NPB	-	-	22-25/25-28	Glass
**[10]**	PEDOT: PSS	Roll-in	ITO/ZnO/PEI/PPV/ MoO_3/PEDOT: PSS	-	5500 (at ~13 V)	91.6	Glass
**[11]**	3ML graphene	Roll-in	ITO/Li:TRE/TRE/(Ir(p py)2(m-bppy)): PGH02/TCTA/TAPC/ HAT-CN/F-MLG/PET	~31.5	~30000 (at ~15 V)	72	Glass
**[12]**	Conducting polymer	Solution method	-	2.8/ 1.3	3200/ 340 (at ~12 V/-)	75	Glass/ PET
**Our Work**	**3L graphene**	**Roll-in**	**ITO/MoO_3/TAPC/Irp py2(acac):$Bepp_2$/Bepp $_2$/Bphen/LiF/TPHP(Li ClO_4)/Graphene**	**44.5**	**9800 (at ~10 V)**	**85**	**Glass**

Supplementary Table 8. Summary of photovoltaic parameters of OSCs with TPHP($CuCl_2$)-coated graphene on PET as anodes under simulated AM 1.5G illumination conditions. PCE in parenthesis is for the best cell.

Anode	J_{SC} (mA cm^{-2})	V_{OC} (V)	FF (%)	PCE (%)
**TPHP($CuCl_2$)-coated graphene**	12.5±0.2	0.70±0.01	53.2±3.4	4.9±0.4 (5.5)

Supplementary Table 9. Summary of the photovoltaic parameters of PSCs with/without TPHP($LiClO_4$) and TPHP($CuCl_2$) under simulated AM 1.5G illumination conditions. PCE in parenthesis is for the best cell.

	†PCE_R/PCE_F (%)	†V_{OC}_R/ V_{OC} _F (V)	†J_{SC} _R/ J_{SC} _F (mA cm^{-2})	†FF_R/FF_F (%)
*w EIL & HIL	19.77/19.62	1.14/1.14	22.40/22.40	77.41/76.84
*w/o	17.50/19.18	1.12/1.13	22.18/22.14	70.44/76.67

*EIL represents TPHP($LiClO_4$) and HIL represents TPHP($CuCl_2$).

†"R" and "F" means reverse and forward scanning, respectively.

Supplementary References

1. Sorel, S., Bellet, D. & Coleman, J. N. Relationship between material properties and transparent heater performance for both bulk-like and percolative nanostructured networks. *ACS Nano* **8**, 4805-4814 (2014).
2. Kim, H.-M. et al. Semi-transparent quantum-dot light emitting diodes with an inverted structure. *J. Mater. Chem. C* **2**, 2259-2265 (2014).
3. Kim, H. Y. et al. Transparent InP quantum dot light-emitting diodes with ZrO_2 electron transport layer and Indium Zinc Oxide top electrode. *Adv. Funct. Mater.* **26**, 3454-3461 (2016).
4. Seo, J. T. et al. Fully transparent quantum dot light-emitting diode integrated with graphene anode and cathode. *ACS Nano* **8**, 12476-12482 (2014).

5. Yao, L. et al. Fully transparent quantum dot light-emitting diode with a laminated top graphene anode. *ACS Appl. Mater. Interfaces* **9**, 24005-24010 (2017).

6. Chen, L. X. et al. Interface dipole for remarkable efficiency enhancement in all-solution-processable transparent inverted quantum dot light-emitting diodes. *J. Mater. Chem. C* **6**, 2596-2603 (2018).

7. Gorrn, P. et al. Towards see-through displays: Fully transparent thin-film transistors driving transparent organic light-emitting diodes. *Adv. Mater.* **18**, 738-742 (2006).

8. Choi, C. S. et al. Blur-free outcoupling enhancement in transparent organic light emitting diodes: A nanostructure extracting surface plasmon modes. *Adv. Opt. Mater.* **1**, 687-691 (2013).

9. Kim, Y. H. et al. Achieving high efficiency and improved stability in ITO-free transparent organic light-emitting diodes with conductive polymer electrodes. *Adv. Funct. Mater.* **23**, 3763-3769 (2013).

10. Kim, N. et al. Highly conductive all-plastic electrodes fabricated using a novel chemically controlled transfer-printing method. *Adv. Mater.* **27**, 2317-2323 (2015).

11. Lim, J. T. et al. Unraveled face-dependent effects of multilayered graphene embedded in transparent organic light-emitting diodes. *ACS Appl. Mater. Interfaces* **9**, 43105-43112 (2017).

12. Kee, S. et al. Highly deformable and see-through polymer light-emitting diodes with all-conducting-polymer electrodes. *Adv. Mater.* **30**, 1703437 (2018).

13. Kim, J. W. and A. Kim. Absolute work function measurement by using photoelectron spectroscopy. *Curr. Appl. Phys.* **31**, 52-59 (2021).
