# Supplementary material for: A polymer electrolyte design enables ultralow-work-function electrode for high-performance optoelectronics
Source: Nat Commun. 2022 Aug 25;13:4987. doi: 10.1038/s41467-022-32651-z (PMC9411633; doi:10.1038/s41467-022-32651-z)
Supplement: Supplementary file 2 — Description of Additional Supplementary Files [file 41467_2022_32651_MOESM2_ESM.pdf]

Description of Additional Supplementary Files

File Name: Supplementary Movie 1

Description: A transparent red QD-LED with a lighting area of 0.4 × 0.4 cm^2 fabricated using ITO bottom anode, graphene top cathode and TPHP($LiClO_4$) EIL. The luminescence from both ITO and graphene sides is very uniform over the lighting area.

File Name: Supplementary Movie 2

Description: A transparent green OLED with a lighting area of 0.4 × 0.4 cm2 fabricated using ITO bottom anode, graphene top cathode and TPHP($LiClO_4$) EIL. The luminescence from both ITO and graphene sides is very uniform over the lighting area.
